# Distinguishing the molecular diversity, nutrient content, and energetic potential of exometabolomes produced by macroalgae and reef-building corals  

**DOI:** 10.1073/pnas.2110283119

**Published:** 2022-01-31

**Authors:** Linda Wegley Kelly, Craig E. Nelson, Daniel Petras, Irina Koester, Zachary A. Quinlan, Milou G.I. Arts, Louis-Felix Nothias, Jacqueline Comstock, Brandie M. White, Ellen C. Hopmans, Fleur C. van Duyl, Craig A. Carlson, Lihini I. Aluwihare, Pieter C. Dorrestein, Andreas F. Haas

**Affiliations:** ^a^Scripps Institution of Oceanography, University of California San Diego, La Jolla, CA 92037;; ^b^Daniel K. Inouye Center for Microbial Oceanography, School of Ocean and Earth Science and Technology, University of Hawaiʻi at Mānoa, Honolulu, HI 96822;; ^c^Collaborative Mass Spectrometry Innovation Center, Skaggs School of Pharmacy and Pharmaceutical Sciences, University of California San Diego, La Jolla, CA 92093;; ^d^CMFI Cluster of Excellence, Interfaculty Institute of Microbiology and Medicine, University of Tübingen, 72076 Tübingen, Germany;; ^e^Department of Biology, San Diego State University, San Diego, CA 92182;; ^f^Department of Microbiology & Biogeochemistry, NIOZ Royal Netherlands Institute for Sea Research, Texel, The Netherlands;; ^g^Department of Ecology, Evolution, and Marine Biology, University of California, Santa Barbara, CA 93106

**Keywords:** coral reefs, metabolomics, molecular networking

## Abstract

Metabolites exuded by primary producers comprise a significant fraction of marine dissolved organic matter, a poorly characterized, heterogenous mixture that dictates microbial metabolism and biogeochemical cycling. We present a foundational untargeted molecular analysis of exudates released by coral reef primary producers using liquid chromatography–tandem mass spectrometry to examine compounds produced by two coral species and three types of algae (macroalgae, turfing microalgae, and crustose coralline algae [CCA]) from Mo’orea, French Polynesia. Of 10,568 distinct ion features recovered from reef and mesocosm waters, 1,667 were exuded by producers; the majority (86%) were organism specific, reflecting a clear divide between coral and algal exometabolomes. These data allowed us to examine two tenets of coral reef ecology at the molecular level. First, stoichiometric analyses show a significantly reduced nominal carbon oxidation state of algal exometabolites than coral exometabolites, illustrating one ecological mechanism by which algal phase shifts engender fundamental changes in the biogeochemistry of reef biomes. Second, coral and algal exometabolomes were differentially enriched in organic macronutrients, revealing a mechanism for reef nutrient-recycling. Coral exometabolomes were enriched in diverse sources of nitrogen and phosphorus, including tyrosine derivatives, oleoyl-taurines, and acyl carnitines. Exometabolites of CCA and turf algae were significantly enriched in nitrogen with distinct signals from polyketide macrolactams and alkaloids, respectively. Macroalgal exometabolomes were dominated by nonnitrogenous compounds, including diverse prenol lipids and steroids. This study provides molecular-level insights into biogeochemical cycling on coral reefs and illustrates how changing benthic cover on reefs influences reef water chemistry with implications for microbial metabolism.

## Significance

Marine dissolved organic matter (DOM) is one of the most complex and abundant chemical mixtures on earth, comprising thousands of different molecules. The molecular structure of these compounds is one factor structuring the community of microorganisms that metabolize them; in turn, this microbial metabolism mediates the composition of DOM. Decades of coral reef research has established the fundamental importance of microbial biogeochemistry in ecosystem function. This study unveils coral reef DOM by identifying a myriad of specific metabolite classes released into the surrounding waters by reef-building corals and algae, further characterizing their energetic and nutrient content and providing a foundation for linking benthic ecology with microbial processes that influence both the livelihood and demise of coral reefs.

Coral reef ecosystems exhibit some of the highest rates of both primary production and organic decomposition across marine and terrestrial environments (1). Benthic primary producers—corals (via their photosynthetic endosymbionts) and algae—impact the concentrations of oxygen, inorganic carbon, and mineral nutrients in the oceanic waters that bathe the reef platform through photosynthesis, respiration, and calcification (2). In addition, these benthic primary producers exude significant amounts of dissolved organic matter (DOM) directly into the surrounding waters (averaging 30% of fixed carbon) (3, 4), establishing the base of a robust microbial food web (5). The molecular diversity of DOM is an essential determinant of biological reactivity; bioavailability of DOM is a continuous function of the interaction between chemical structure and the metabolic capacity of the microbial community in an environment (6). Microorganisms comprise the majority of biomass in the water column of oligotrophic oceans where most euphotic reefs persist, and their role in the decomposition of dissolved organic substrates is critical to nutrient-recycling as well as for channeling nutrients and energy into the food web (7, 8). Reefs are increasingly recognized as fundamentally microbial ecosystems, with microbially mediated symbioses and biogeochemical cycling playing key roles in function and resilience (9–11). Thus, characterizing the quantity and composition of DOM produced by corals and algae in reefs is crucial for understanding the microbial processes that sustain coral reef functions.

Contrasting the composition of DOM released by specific taxonomic groups of corals and algae is important for understanding long-term global trends toward reef degradation and increases in fleshy algal dominance (12, 13). Two recent studies used high-performance liquid chromatography–mass spectrometry (LC-MS) to apply a combination of targeted and untargeted metabolomics for characterizing the composition of DOM associated with coral reef ecosystems; Fiore, Freeman, and Kujawinski (2017) described sponge transformation of coral reef DOM, while Weber and colleagues (2020) conducted an extensive comparison of reef DOM composition across Caribbean coral reefs (14, 15). Going forward, characterizing the complexity of discrete sources of DOM to these complex pools is necessary to understand how DOM is cycled in these hyper-diverse ecosystems. Empirical studies on coral reefs have demonstrated that rapid and sustained release of labile DOM by fleshy algae supports elevated microbial growth with corresponding destabilization of the community composition and shifts in the rates and efficiencies of organic matter metabolism by bacterial communities (3, 4, 16, 17). One mechanism that may drive these fundamental differences in microbial metabolism is the broad divergence in the types of molecules and energetic potential of DOM released from different benthic producers. Prior observations suggest that microbial activity is greater at degraded reef sites with higher coverage of fleshy algae (16); one possible mechanism promoting this process is more available energy in the DOM pool. Molecules with lower oxidation states provide more potential energy per carbon during catabolism (18). We hypothesize that the chemical composition, macronutrient content, and carbon oxidation state potential of DOM exuded by different reef primary producers is distinct and modulates how shifts in benthic community structure fundamentally alter the recycling of carbon and other nutrients in reef ecosystems (19), with implications for energy transfer to higher trophic levels (20). To this end, examining both the stoichiometric and structural characteristics of the distinct suites of metabolites released by different coral reef organisms is a promising avenue for identifying chemical classes and metabolic pathways that drive changes in coral reef ecosystem function.

The compositional complexity of marine DOM challenges our ability to fully resolve production and consumption processes in the ocean. Defining both stoichiometric composition and structural classes of DOM are central to predicting microbial metabolic processes. Current analytical approaches (isolation and characterization) are overwhelmed by the chemical diversity of DOM. For example, less than 10% of the potentially relevant compounds can currently be identified by matching to existing compound libraries likely due to both the limited representation of marine metabolites in current libraries and data quality that is compromised by sample complexity (14). Furthermore, measuring ecologically relevant changes in the concentrations of specific compounds often exceeds instrument precision (21). Hardware advances of high-resolution tandem mass spectrometers used for untargeted metabolomics are certainly increasing our ability to detect rare and diverse compounds semiquantitatively (e.g., refs. 22 and 23). Rapid advances in computational tools have also been crucial for moving into the realm of dark metabolomics (24), where elemental composition and compound class-level information can be assigned to detected features with tandem fragmentation spectra with increasing confidence (22, 25–27).

To better understand reef metabolism at the biochemical level, we examined both the bulk elemental characteristics and features of organic exudates released both daytime and nighttime by five different benthic primary producers in the coral reefs of Mo’orea, French Polynesia: reef-building corals (genera *Pocillopora* and *Porites*) and representatives of three dominant functional groups of algae: crustose coralline algae (CCA), the fleshy macroalga *Dictyota*, and turfing microalgae. We isolated a fraction of DOM (roughly 25%) from reef and incubation water via solid-phase extraction (SPE) (28) and subjected 42 samples to liquid chromatographic separation with positive electrospray ionization Orbitrap-based tandem mass spectrometric detection (LC-MS/MS) of unique mass-to-charge ratios and retention times (MS1 features) (23). The corresponding fragmentation spectra (MS2) were analyzed via Global Natural Product Social Molecular Networking (GNPS) (21, 22), a software ecosystem that integrates several emerging tools in untargeted tandem mass spectrometry. Informatic tools incorporated in the GNPS workflow allow for feature detection and alignment from extracted ion chromatograms (XICs) across the entire dataset to collapse identical features (29), consensus MS2 spectral generation to minimize chimeric spectra, molecular formula assignment (25, 30), structural annotation, and spectral similarity-based molecular networking (22, 26, 27, 31). Our goal was to resolve suites of chemical classes specific to each producer as well as characterize differences in elemental stoichiometry and redox potential of a fraction of exometabolite pools that may explain the role of coral reef DOM in microbial nutrient-recycling and previously observed shifts in microbial metabolic pathways with increasing algal dominance (16, 32). Our results demonstrate distinct exometabolome features released by each benthic producer, with broad stoichiometric and compound class distinctions between corals and algae, including evidence for differences in carbon oxidation state and organic nutrient content.

## Results

### Organismal Incubations and Sampling.

This study was conducted at the Richard B. Gump South Pacific Research Station in Mo’orea, French Polynesia, in September 2017. To collect exudates of benthic primary producers triplicate specimens of five common primary producers from different functional groups were collected and acclimated for several days in water tables: the hermatypic zooxanthellate scleractinian corals *Porites lobata* and *Pocillopora verrucosa*, one species of rhodolithic CCA *Hydrolithon reinboldii*, the macroalga *Dictyota ceylanica*, and a typical mixed consortium of benthic turfing algae attached to dead coral rubble (Turf). Specimens were then incubated in 0.2 μm filtered reef water in separate 1.2 L polycarbonate aquaria first over an 8-h daytime period and then, after water replacement, a subsequent 8-h nighttime period. Starting reef water was sampled before incubation (ambient) and reef water without a benthic producer was incubated in parallel in triplicate (Control). A total of 42 water samples were collected for each parameter, including start (n = 3) and end (n = 3) from each of six treatments each repeated in separate daytime and nighttime incubations. Each water sample was subsequently partitioned for analysis of bulk inorganic and organic elemental chemistry (dissolved oxygen [DO], pH, nitrate+nitrite, phosphate, ammonium, silicate, total dissolved nitrogen, total dissolved phosphorus, and dissolved organic carbon), and a fraction of DOM was extracted for characterization using a solid-phase sorbent: PPL (Priority PolLutant), currently the most-widely used method for extracting DOM from seawater (23, 28, 33). Additional methodological details are found in *SI Appendix*.

### Producer Effects on Bulk Elemental Reef Water Biogeochemistry.

**Fig. 1.**
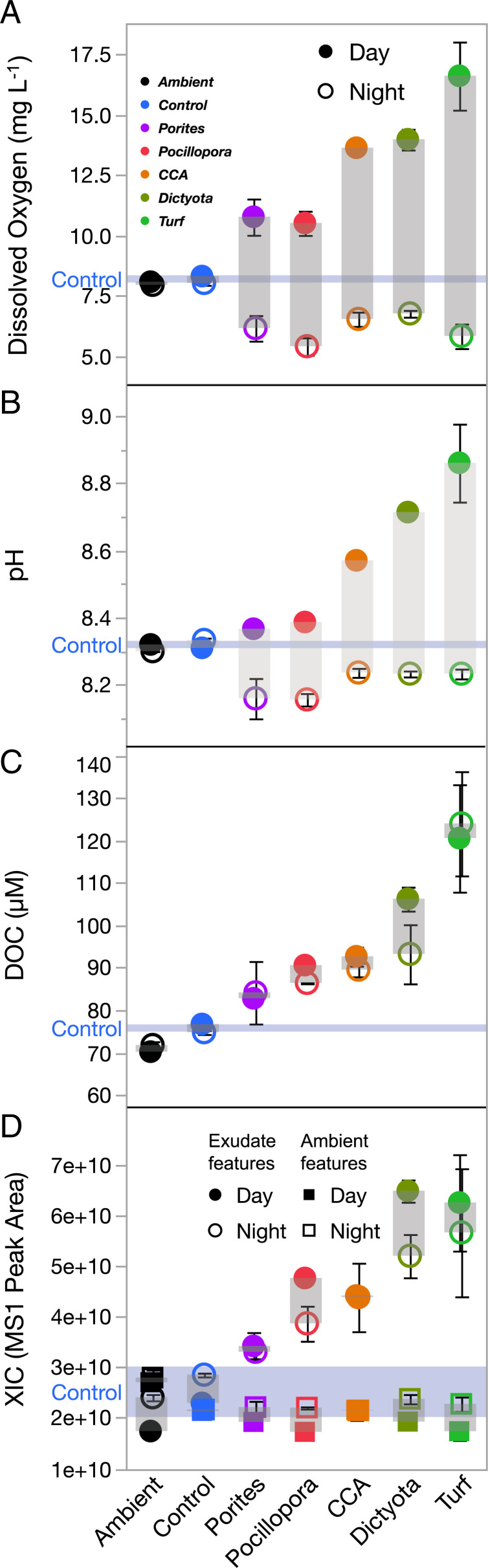
Production/consumption of oxygen, pH, DOC (*A–C*), and summed metabolites during incubations. Symbols are means (± SEM) of triplicate incubations of benthic producer specimens (colors) measured after sequential 8 h day or night incubations (solid or open symbols) (*D*). Water Controls are annotated with a blue line (mean ± SEM). *D* shows XIC MS1 peak areas summed for all features divided into Exudate features (circles; those increasing at least twofold in any treatment over the 8-h incubation) and Ambient features (squares; those with less than twofold change from ambient water at the start of the experiment).

The benthic primary producers exhibited diurnal impacts on water chemistry parameters, such as DO concentrations, pH, and nutrient availability, consistent with previous observations (4, 34, 35). The chemistry among endpoint incubations was compared both day and night to determine which primary producer treatments significantly altered water chemistry parameters relative to the incubated seawater Controls (changes in water chemistry of Controls over the 8-h incubations were minor and generally not significant; *SI Appendix*, Fig. S1). Dissolved oxygen, pH, and dissolved organic carbon (DOC) differed significantly from the Controls and among the five producer treatments in both daytime and nighttime incubations (false discovery rate [FDR]–adjusted ANOVA *P* < 0.05; Fig. 1 *A–C*, respectively). In daytime incubations, DO concentrations and pH were significantly higher than the endpoint Control in all algal treatments (Turf, Dictyota, and CCA; Dunnett’s *P* < 0.02; Fig. 1 *A* and *B*). At night, oxygen was depleted in the turf algae treatments relative to the Controls, and oxygen depletions were accompanied by decreased pH values in both coral species incubations (Dunnett’s *P* < 0.02; Fig. 1 *A* and *B*).

All organisms released significant quantities of DOC into the surrounding water both day and night, ranging from 6 to 10 µM (*Porites*) to 44 to 49 µM (Turf) above the Controls. DOC was significantly enriched in both noncalcifying algae (Turf and *Dictyota*) during the day, but only Turf treatments had significantly higher DOC concentrations during the night incubations relative to the Controls (Fig. 1*C*; Dunnett’s *P* < 0.01). DOC concentration was strongly positively correlated with concentrations of oxygen and pH during daytime incubations (R^2^ > 0.8, *P* < 0.0001; *SI Appendix*, Fig. S2 *A* and *B*) but independent of oxygen concentration and pH during nighttime incubations (*P* > 0.05; *SI Appendix*, Fig. S2 *F* and *G*), suggesting that both daytime and nighttime DOC release rates are determined by daytime photosynthesis.

Total dissolved phosphorus and nitrogen concentrations showed distinct patterns for each organism in daytime incubations and were enriched relative to the Controls, with both coral taxa enriched in phosphate and all taxa enriched in either or both dissolved inorganic or dissolved organic nitrogen (DON) (*SI Appendix*, Fig. S3). Specific dissolved nutrients (PO_4_^3−^, NO_3_^−^+NO_2_^−^, NH_4_^+^, DOP [dissolved organic phosphorus], and DON) differed significantly among treatments only during daytime experiments (*SI Appendix*, Fig. S3; FDR-corrected ANOVA *P* < 0.01; silicate did not differ from Controls). Relative to Controls, *Pocillopora* incubations were significantly enriched in all five nutrients, with PO_4_ additionally enriched in *Porites* incubations and DON additionally enriched in *Porites*, CCA, and Turf algal incubations (Dunnett’s *P* < 0.05). Compared with the ambient water, both coral taxa enriched seawater nitrate, and all three algae depleted phosphate (Dunnett’s *P* < 0.05; *SI Appendix*, Fig. S3).

Previous work has shown different spectral classes of fluorescent DOM to be released autochthonously from macroalgae and corals (36), and our measurements corroborated this finding with algae enriched in humic-like components and humification indices and corals enriched in proteinaceous components such as tyrosine- and tryptophan-like fluorescence (*SI Appendix*, Fig. S4*A*). We found significant daytime enrichment of all dominant fDOM components in all five primary producer exudates relative to both the Ambient and Control water incubations, which did not differ significantly (*SI Appendix*, Fig. S4*B*). Only the tyrosine-like FDOM exhibited a clear day–night interaction, with significantly less enrichment by coral exudates in the nighttime incubations. Filtration reduced cell concentrations 10-fold at the start of incubations (to roughly 5 × 10^4^ cells mL^−1^ in the water Controls at the start of the experiment; Controls increased roughly threefold to 1.5 × 10^5^ cells mL^−1^ over the course of the experiment while benthic producer treatments ranged from two to three times Controls due to a combination of growth and cells introduced with the producers, never exceeding the ambient cell concentrations in the reef [roughly 5 × 10^5^ cells mL^−1^]).

### Tandem Mass Spectrometry Feature Abundance in DOM.

Using untargeted tandem mass spectrometry, a total of 20,742 unique mass-retention features were extracted from a pool of 258 field-collected and mesocosm experiment samples from Mo’orea analyzed together via LC-MS/MS. The 42 exometabolome samples in this dataset were combined with 208 additional environmental samples (collected from adjacent reef waters and incubation experiments conducted in Mo’orea within a week of this experiment) and eight daily process blank samples (Fisher Optima LC/MS grade water run through all equipment) for a total of 258 samples analyzed in one sequential batch of a liquid chromatography–tandem mass spectrometry run; using a larger sample set from a single ecosystem improves consensus alignment of MS2 fragmentation spectra which improves database matching and improves resolution of system “background features” resolved from method blanks (“Statistical Analyses”). From these features, 6,899 were removed as background features (features recovered in eight method blanks), and an additional 3,275 were removed as transient features (features replicated in less than three of the 42 exudate samples examined in the present study) for a final dataset of 10,568 common features with tandem fragmentation mass spectra. From here, features were differentiated as either newly produced DOM (8,936 “exudate” features: those with at least twice the XIC peak area in any incubation treatment, including Controls, relative to the ambient starting water) or as ambient DOM (1,632 “ambient” features: those never twice the mean peak area of ambient seawater over the 8-h incubation). Importantly, the summed ion peak areas of exudate features in each sample increased with DOC release whereas summed peak areas of ambient features were stable across all sample types (Fig. 1*D*), indicating that the quantity of ion features covaried with the independently measured quantity of DOC release (Fig. 1*C*). Moreover, there was a strong and significant correlation between the summed exudate ion peak areas and DOC concentration both in daytime (R^2^ = 0.84) and nighttime (R^2^ = 0.63) incubations (*SI Appendix*, Fig. S2 *D* and *I*) demonstrating that despite likely extraction and ionization biases, the abundance of these features tracked total DOC concentration indicating that a comparable fraction of the total exuded DOM was resolved by the mass spectrometry analysis among treatments. There was no significant correlation in either day or night incubations between ambient feature summed ion peak areas and DOC (*SI Appendix*, Fig. S2 *E* and *J*), suggesting that these features comprise DOM present in the ambient seawater that is not produced by any of the experimental organisms in this study.

### Discrimination of Benthic Producer Exometabolite Features.

Of the 8,936 exudate features, a subset of 1,667 was statistically significantly enriched in at least one of the five organismal incubations relative to the Control aquaria at the end of daytime and/or nighttime incubations (ANOVA with Dunnett’s post hoc FDR-adjusted *P* < 0.05) (37). On average, these features were 40-fold enriched relative to Controls (SD fivefold) and were defined as a statistically conservative suite of unambiguous benthic primary producer exometabolite features. Of the 1,346 exometabolite features enriched in daytime incubations, the vast majority (86%; 1,158) were uniquely significantly enriched in only one of the five treatments (ranging from 31 unique features in *Porites* to 382 features in *Pocillopora*; *SI Appendix*, Fig. S5), and an additional 141 in just two of the five; only three exometabolite features were enriched in every primary producer incubation (*SI Appendix*, Fig. S5). Summed together, exometabolite features comprised on average 8 to 24% of the total peak area in benthic producer incubations. A small proportion of these exometabolite features (∼4% of total peak area) were measured in both starting ambient and ending Control water (*SI Appendix*, Fig. S6), which we interpret to represent benthic producer-derived exudates persisting in the reef waters. Of the remaining 7,269 exudate features not meeting the stringent statistical criteria for exometabolite features, 2,047 planktonic exudate features (doubled in Controls) averaged 21 to 28% of total sample peak area and 4,846 benthic exudate features (doubled in organismal incubations) averaged 22 to 33% of total peak area in incubations (*SI Appendix*, Fig. S6).

### Compositional Differences among Benthic Producer Exometabolomes.

**Fig. 2.**
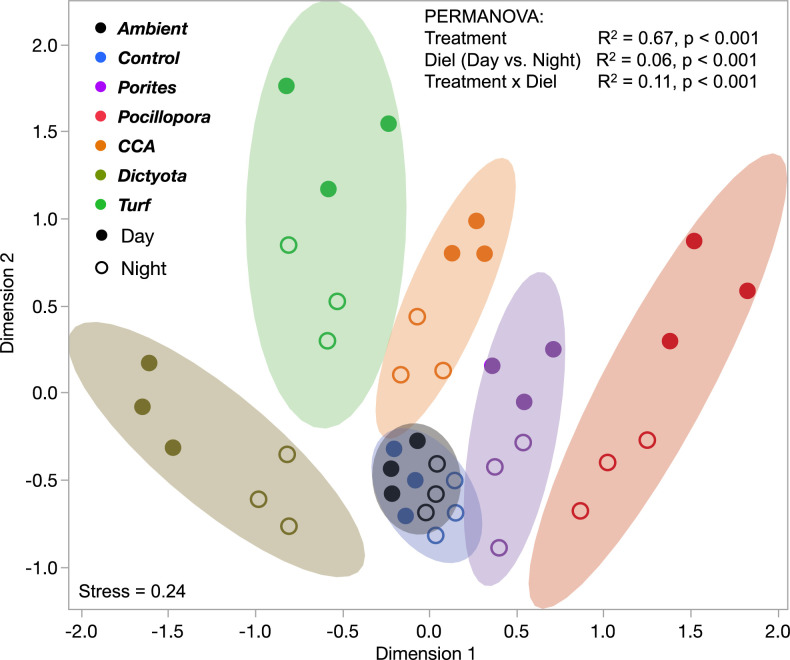
Ordination of daytime and nighttime incubation samples according to relative abundance of exometabolite features. Bivariate normal density ellipses contain 90% of the data. Relative abundances of each exometabolite feature (n = 1,667) in each sample were angular transformed to approximate Gaussian distributions and then standardized via z-scoring before ordinating via multidimensional scaling (aka principal coordinates analysis). Results of permutational ANOVA via the adonis function in the vegan package in R are presented at top. All treatment pairwise comparisons are *P* < 0.01 except Ambient-Control *P* = 0.13.

The relative abundances of exometabolite features differed clearly and significantly among each of the five benthic producers and Controls: we observed a clear separation between the different exometabolomes, with a gradient from fleshy algae (turf and macroalgae) through CCA to corals along the first dimension of multidimensional scaling ordination (Fig. 2). Light availability influenced exometabolome composition as well, with increased differentiation of exometabolomes from different benthic producer sources and increased differentiation from the Control in daytime relative to nighttime (Fig. 2). Exometabolite features were clearly associated with specific benthic producers, forming distinct modules according to whether they were exuded at all times or solely during the daytime; fewer features were primarily associated with nighttime exudation (*SI Appendix*, Fig. S7). Benthic exometabolomes differed strongly by primary producer (Fig. 2; R^2^ = 0.67, *P* < 0.0001) with smaller differences between day and night exudations (R^2^ = 0.06, *P* < 0.0001) and diel–treatment interactions (R^2^ = 0.11, *P* < 0.0001). All treatment and diel categories differed significantly (pairwise *P* < 0.01) except the Ambient and Control treatments (*P* = 0.13; Fig. 2); *SI Appendix*, Fig. S8 summarizes the results of pairwise treatment tests. The relatively small compositional differences observed between day and night samples for each organism (Fig. 2 and *SI Appendix*, Fig. S8) are consistent with only subtle diel differences in quantities of DOM exuded (Fig. 1 *C* and *D*). Day and night incubations differed markedly in pH and DO (Fig. 1 *A* and *B*), but these swings did not appear to impact physiology in a manner that changes the composition of exudates: mean organism diel compositional differences were not different from the diel shifts in the water Controls (which exhibited no differential nighttime deoxygenation/acidification; *SI Appendix*, Fig. S8). Because 81% of exometabolite features associated with any benthic producer treatment were produced in the daytime, the small proportion of night-only exometabolite features was excluded from subsequent analyses to simplify the presentation of exometabolite stoichiometry and feature structural classifications, although exploring nighttime exudation dynamics in laboratory, field mesocosm, and survey contexts is a priority for future work.

### Energetic Content of Benthic Producer Exometabolomes.

**Fig. 3.**
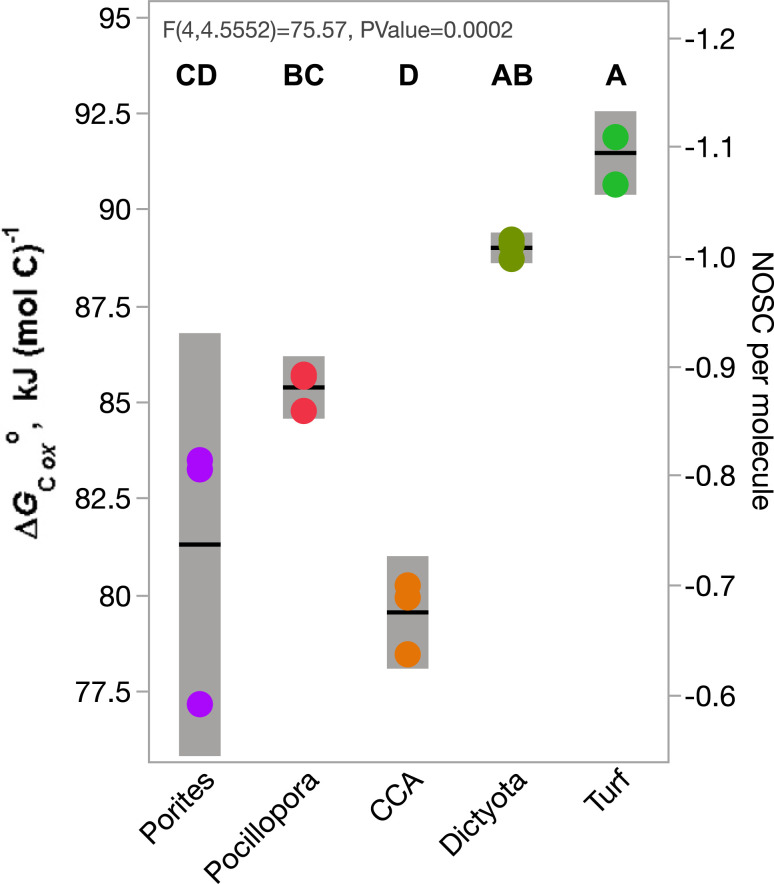
Gibbs energy and NOSC in exometabolite samples of coral reef benthic producers. Gibbs energy (*Left* primary axis) derived from the weighted mean NOSC (*Right* secondary axis) in triplicate exometabolite feature samples of five organisms during daytime incubations. Whiskers are SEs of means and treatments sharing letters at the *Top* are not significantly different at Tukey post hoc *P* < 0.05.

Calculating the average nominal oxidation state of carbon (Z_C_ or NOSC) of molecules provides a proxy for estimating the potential energy gained during catabolism (defined as ΔG°C_ox_, the standard molar enthalpy change of combustion at 298 K) (38). The NOSC in daytime exometabolite features differed significantly among treatments (*P* < 0.001; Fig. 3) and was significantly higher in fleshy algae (turf and macroalgae, mean estimated ΔG°C_ox_ 89 to 91 kJ/mol/C^−1^) than in calcifying organisms (CCA and corals, mean estimated ΔG°C_ox_ 80 to 85 kJ/mol/C^−1^) (Fig. 3), indicating that the chemical makeup of fleshy algal exometabolomes may provide greater catabolic energy potential than those of calcifying algae and coral. The low NOSC in both algae exometabolomes was due to significantly lower O:C ratios (∼0.15) relative to corals and CCA (0.2 to 0.3) coupled with elevated H:C ratio (>1.2) (*SI Appendix*, Fig. S9), suggesting enrichment in lipid-like DOM. Daytime exometabolomes were distinct among the five incubation treatments in Van Krevelen space (H:C and O:C; *SI Appendix*, Fig. S10). NOSC did not differ among treatments in nighttime exometabolomes (*P* > 0.05).

### Elemental Stoichiometry of Benthic Producer Exometabolomes.

**Fig. 4.**
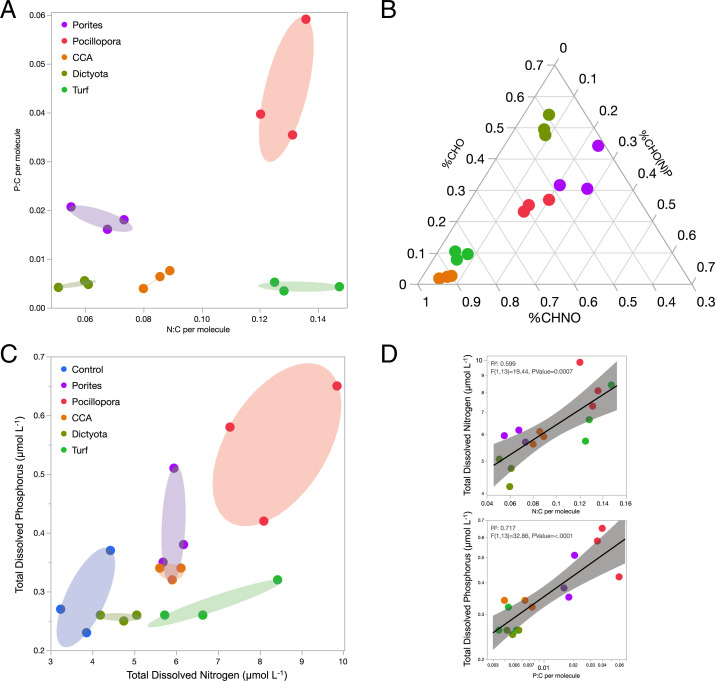
Exometabolite feature N and P stoichiometry predicts bulk N and P release by benthic producers. (*A*) Weighted mean N:C and P:C per molecule mapped among triplicate daytime exometabolite feature samples is coordinated with the (*B*) Ternary plot of proportional weighted mean relative abundance of three stoichiometric classes of exometabolite features. (*C*) Total dissolved N and P (micromolar) released from primary producers in triplicate daytime incubations is coordinated with the organic nutrient stoichiometry of exometabolite features. (*D*) The weighted average N:C and P:C of exometabolite features (from 4*A*) predict the concentrations of N and P (from 4*C*), respectively.

Macronutrient content of DOM can play a central role in how microbial remineralization processes contribute to reef productivity and nutrient retention. Algal and coral exometabolomes differed significantly in macronutrient stoichiometry (Fig. 4 *A* and *B*): coral exudates were enriched in both bulk P content and the relative abundance of molecules containing P, exudates from the macroalga *Dictyota* were depleted in both N and P, while *Pocillopora*, Turf, and CCA exudates contained more N per molecule and had a higher proportion of molecules containing N than the other treatments. The stoichiometric patterns of the producer exometabolomes were reflected in the bulk water column elemental concentrations (Fig. 4*C*), with *Dictyota* exudation only slightly enriched in N relative to the Controls, P release highest in corals, and N release highest in Turf and *Pocillopora*. Exometabolome stoichiometry covaried with bulk elemental measurements of dissolved nutrients, with weighted mean exometabolite feature N:C predicting total dissolved nitrogen concentrations and weighted mean exometabolite feature P:C predicting total dissolved phosphorus concentrations in the water (Fig. 4*D*). These stoichiometric patterns were generally visible in raw exometabolite elemental content (*SI Appendix*, Fig. S11 *A*, *B*, *D*, and *E*) and were consistent across exudate N and P in bulk organic and inorganic compounds (*SI Appendix*, Fig. S11 *C* and *F*).

### Dominant Compound Classes Found in Benthic Producer Exometabolomes.

**Fig. 5.**
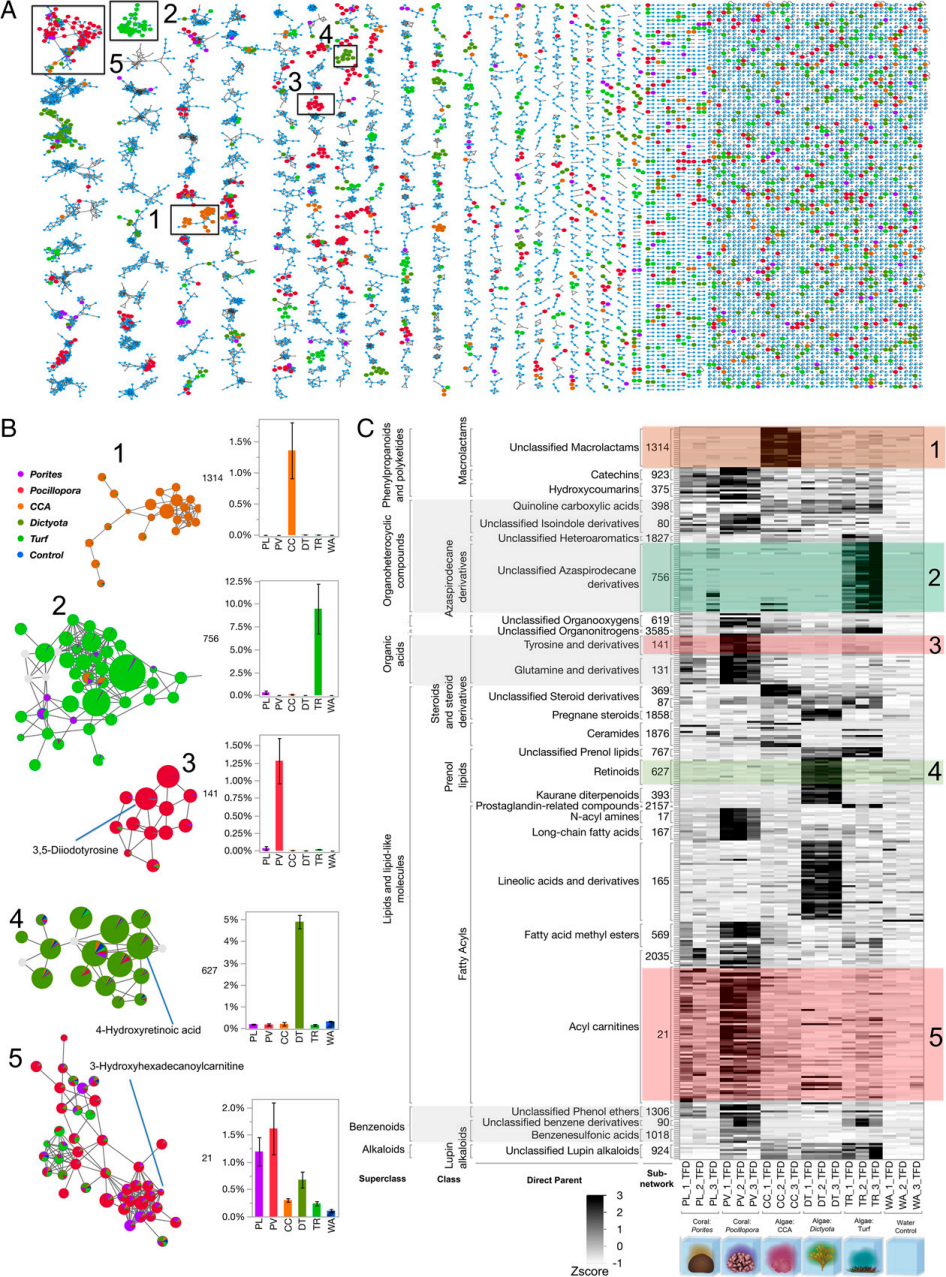
Distinct chemical classes of exometabolite feature subnetworks enriched in specific primary producers. (*A*) Spectral network of all MS1 ion features (nodes) linked by spectral cosine scores >0.7 (edges). Selected subnetworks significantly enriched in specific treatments are boxed (1–5) and shown in *B* with exact library matches annotated and barcharts of summed node relative abundance of subnetworks across treatments. Nodes are sized by the log of the mean MS1 peak areas in the highest treatment and colored by the mean proportion of total MS1 peak area from each treatment. (*C*) Heatmap of standardized (z-score) relative abundance of 29 subnetworks (numbered per 5*B*) comprising 361 metabolite features (rows) across Daytime endpoint treatments with ClassyFire ontology annotations (*SI Appendix*, Fig. S12).

The exudate features released by each benthic producer showed clear patterns of structural specificity (Fig. 5). Molecular networking (GNPS) (22) organized all 10,568 common features into molecular families (subnetwork clusters); when features network together, their MS2 fragmentation spectra match inferring structural relatedness (Fig. 5*A*). Most subnetworks containing features that had been classified as exometabolites by the statistical tests were observed to be producer specific: out of 430 subnetworks containing exometabolite features, 291 subnetworks were statistically enriched by at least twofold in one or more benthic producer treatment (Dunnett’s *P* < 0.05 following FDR), 63% of which were enriched in just one of the five producer treatments (*SI Appendix*, Table S1). Some of these producer-specific exometabolite subnetworks represented a substantial proportion of the total peak area in each treatment. For example, Turf and *Dictyota* exuded suites of related molecules that comprised a mean of 9% and 5% of the total peak area, respectively (subnetworks 756 and 627; Fig. 5*B*). Finally, subnetworks hierarchically grouped by broad chemical classifications illustrate patterns of preferential exudation of specific compound classes among the five reef primary producers (Fig. 5*C*). Producer-specific exudates were visualized as a subset of feature networks that were all abundant (mean summed relative abundance greater than 0.05% of at least one exometabolome), diverse (greater than five exudate nodes in a subnetwork), and significantly enriched (summed mean more than twice the Control relative abundance and statistically enriched as tested by Dunnett’s FDR *P* < 0.01). The standardized relative abundance and classification of the 361 features comprising these 29 most-abundant networks is shown in Fig. 5*C*. Manually verified structural annotations of 17 of those networks containing nodes that were spectral matches to compounds in major databases are shown in *SI Appendix*, Fig. S12; the remaining subnetworks did not contain nodes with spectral library matches and were broadly classified via the MolNetEnhancer in silico structural consensus prediction tool (27).

Both coral genera produced numerous exometabolite features identified as acyl carnitines that comprised 69 distinct spectral features and collectively summed 1.9% of the total peak area in *Pocillopora* treatments (subnetwork 21; Fig. 5). Additionally, both corals released 10 other subnetworks (*SI Appendix*, Table S1), including exometabolite features annotated as isoindoles (subnetwork 80) and catechins (subnetwork 923; Fig. 5*C*). A total of 22 subnetworks (*SI Appendix*, Table S1) were released by both types of fleshy algae, Turf and *Dictyota*, including exometabolite features broadly classified at heterocyclic compounds (subnetwork 398). There were also 12 subnetworks significantly released by all three algal taxa (*SI Appendix*, Table S1), including a suite of exometabolite features broadly annotated as alkaloids (subnetwork 924; Fig. 5*C*). Most exometabolite features and subnetworks (63%) were uniquely produced by only one of the organisms studied in this experiment; here, we describe a few noteworthy examples. *Pocillopora*, with a total of 382 unique significant exometabolite features, was the only producer significantly enriched in 65 subnetworks, which included the acyl carnitines (subnetworks 21 and 2,035) and other distinct fatty acyls (subnetworks 167 and 569), but also two distinct suites of features annotated as organic acids: halogenated tyrosine derivatives (subnetwork 141) and oleoyl-taurine (subnetwork 17) (Fig. 5 *B* and *C*). CCA produced 20 distinct subnetworks that included a cluster of 25 spectrally related exometabolite features annotated as nitrogen-containing polyketide macrolactams (subnetwork 1,314; Fig. 5 *B* and *C*). The most-significant suite of compounds released by Turf was classified as nitrogen-containing heterocyclic compounds structurally similar to alkaloids (subnetwork 756; Fig. 5 *B* and *C*). *Dictyota* released the second-highest number of unique exometabolite features (325) with 52 distinct networks. The most-notable molecular classes were identified as distinct clusters of lipids (subnetworks 165 and 627, Fig. 5*C*). Subnetwork 165 was represented by 40 exometabolite features including four library matches identified as lineolic acids that could make up 4% of the total peak area. Subnetwork 627 comprised 14 features with library matches identified as 4-hydroxyretinoic acid, which represented a significant proportion of the total peak area in *Dictyota* treatments (up to 5%; Fig. 5*B*). These molecular differences in primary producer exometabolite features will have significant implications for organic nutrient-recycling in the generally oligotrophic waters of coral reef environments.

## Discussion

The results of this study begin to unlock the black box of DOM composition in a well-studied coral reef ecosystem (17, 39), providing mechanistic insights into the role of DOM in coral reef microbial ecology and biogeochemistry. Our results demonstrate unequivocally that a broad array of coral reef primary producers each release exudates of distinct chemical classes and with specific chemical stoichiometries in significant quantities—both day and night. Based on structures (spectral library match annotations) and compound class identities (in silico annotations) determined in this study, each reef producer has the capacity to differentially influence the nutrient availability and energetic content of the DOM pool. In addition, the patterns in multivariate exometabolite feature variation among producers, including enrichment of P in coral exometabolite features, enrichment of N in turfing algal exometabolite features, and evidence of highly condensed and reduced features released primarily by fleshy algae (Figs. 3 and 4 and *SI Appendix*, Figs. S3, S4, and S9–S11), are consistent with our observations of bulk DOM stoichiometry (DOC, DON, and DOP), inorganic nutrients, and fluorescent characterization of DOM. In terms of chemical diversity, our results highlight the structural similarities of exometabolite features, belonging to narrowly characterized chemical classes, that are specific to each benthic producer and contribute to these patterns in exometabolome biogeochemistry (Fig. 5).

### Untargeted Metabolomics Can Characterize and Distinguish Biogeochemically Relevant Fractions of DOM.

Our results outline a methodological approach to compositionally distinguish thousands of ambient DOM features from those freshly produced to specifically identify benthic primary producer exometabolite features (Figs. 1 and 2 and *SI Appendix*, Figs. S4–S8). This contribution alone can facilitate examination of the distribution and abundance of both these types of features in reefs, eventually enabling the comparison of field (e.g., ref. 15) with experimental studies to evaluate the lability and persistence of different chemical classes of DOM. Ambient features dominated the DOM pool in the open reef waters (57% of ion peak area), while planktonic exudate features were just 14% (*SI Appendix*, Fig. S6), likely reflecting the diversity of semilabile or refractory marine DOM, which is not metabolized on time scales comparable to the seawater reef residence time of hours to days (40). After 8 h of incubation in filtered seawater, these ratios balanced to 46% and 29% of metabolites, respectively, suggesting that roughly 10 to 15% of this metabolite pool is in flux via planktonic exudation and/or microbial remineralization and transformation. Benthic exometabolite features comprised 8 to 24% of the feature pool (*SI Appendix*, Fig. S6) but only 4% in both starting and ending water Controls, reinforcing the hypothesis that many of these features are labile on relatively short timescales within the reef ecosystem, while a small proportion is found in the ambient water and is therefore potentially semilabile or recalcitrant to microbial degradation. Certainly, these methods provide an opportunity to further define the production and remineralization of benthic and phytoplankton exudates across coastal systems. By demonstrating that various benthic producers release SPE-extractable DOM with distinct oxidation states (Fig. 3 and *SI Appendix*, Figs. S9 and S10), macronutrient stoichiometry (Fig. 4 and *SI Appendix*, Figs. S3 and S11), and chemical class compositions (Fig. 5), this work tackles two central questions in marine DOM biogeochemistry (41): resolving key sources of prototypical molecules in the reef DOM pool and addressing how element cycles can be linked through marine DOM.

### Characterization of DOM in Reefs Provides Insight into the Biogeochemical Impacts of Local and Global Changes in Reef Cover and Nutrient Enrichment.

By illuminating some chemical characteristics of benthic exometabolomes released into coral reefs (Figs. 2 and 5), this work provides a foundation upon which a mechanistic understanding of how chemical diversity and complexity in coral reef environments influence biogeochemistry (5). Recent work has established that corals and benthic algae exude significant concentrations of newly synthesized DOM that serve as rich substrates for microbial growth and remineralization that influence local ecosystem processes such as the dynamics of oxygen and pH (3, 4, 17, 36, 42). However, this study has revealed the structural classifications and stoichiometries of a fraction of the exudate compounds driving these dynamics. In addition, by relating the stoichiometry of exometabolite features to bulk nutrient fluxes (Fig. 4*D*), this work links exometabolome composition to processes such as the microbialization of coral reefs due to phase shifts to algal dominance (16) and the macronutrient-recycling processes that help maintain reef productivity in nutrient-depleted tropical oceans (43). Differences in the molecular composition of bioavailable DOM from various benthic and pelagic sources may control some of the observed variation in resident microbial communities observed at different reef environments (17, 39).

While our elemental analyses represent the bulk organic carbon, nitrogen, and phosphorus content of exudates thoroughly (Figs. 1 and 4 and *SI Appendix*, Figs. S2, S3, and S11), any mass spectral analysis of solid-phase extractable compounds can only characterize a fraction of the DOM pool dictated by biases of extraction and ionization, ascribing a particular analytical window to our molecular analyses of exometabolite features. Our extraction efficiencies of exudates ranged from 21 to 26%, which is typical for freshly produced DOM (44), and did not differ significantly among treatments in either day or night (*P* = 0.075 and *P* = 0.084, respectively; *SI Appendix*, Fig. S13). While aromatic and larger nonpolar, uncharged compounds are well retained on PPL, there is evidence of low retention efficiencies for carbohydrates, proteins, and peptides (45, 46), but bioavailable components of the marine DOM pool are retained on PPL (44). Variation among extracted compounds in ionization potential, ionization suppression, and successful MS2 fragmentation prevents assignment of even rough estimates of the proportion of extracted DOM captured in the mass spectral analyses (21, 47). Nonetheless, we coordinated a suite of statistical and analytical rigorous tests to demonstrate that the results derived from the fraction of DOM that we are analyzing are robust, including demonstrating that the fraction scales with bulk DOC (Fig. 1 and *SI Appendix*, Fig. S2) and represents a significant proportion of the total ion peak area (*SI Appendix*, Fig. S6) and further demonstrating that the fraction comprises thousands of compounds (*SI Appendix*, Fig. S5) that are distinct among different sources in relative abundance (Fig. 2 and *SI Appendix*, Fig. S7), elemental composition (Fig. 4 and *SI Appendix*, Figs. S9 and S10), and chemical structure (Fig. 5). Our resulting analysis therefore represents a thorough and representative characterization of the fraction of DOM visible using our current state-of-the-art analytical and bioinformatic approaches to untargeted metabolomics.

There is additional potential for differential ionization efficiencies among compound classes, with the potential for ion peak area intensities to poorly represent compound abundances. To evaluate the potential impact of this on our conclusions, we added a supplemental comparison of the results of Fig. 3 using ion feature–standardized scoring: each feature is internally z scored (subtract feature mean peak area and divide by feature SD), eliminating among-feature differential absolute and relative feature abundances to avoid skewing weighted averages toward strongly ionizing compounds. This alternate analysis (*SI Appendix*, Fig. S14) shows that weighted mean NOSC shifts only slightly and many of the relationships between the treatments are preserved, such that within our analytical window, the bias of algal exudates toward more reduced and energetically rich macromolecules is robust.

### Energetic Differences in DOM Exudates Have Ramifications for Reef Ecosystem Microbiology.

The observed differences in the oxidation potential and energetic content of coral and algal exometabolites in this study are consistent with much previous work demonstrating chemical differences in the DOM released by these functionally distinct macroorganisms, many of which have broader analytical frames of reference. Differences in DOC exudates between reef-building corals, CCA and fleshy algae are reflected in bulk concentrations, monosaccharide and carbohydrate composition, and bioavailability (3, 48, 48–50). Nelson et al. demonstrated differences in dissolved combined neutral sugar content, a small but quantitative proportion of the DOM pool (17). Quinlan et al. demonstrated differences in fluorescence characteristics, an operationally defined but poorly constrained subset of DOM (35). Within this study, we demonstrate differences in bulk nutrient content of the DOM, a technique that is quantitative and agnostic to chemical structure. Finally, the statistical differences in estimated energetic content and nutrient content are consistent with independent analyses of differences in the molecular structures observed in algal and coral exudates (Fig. 5), which do not rely on elemental formula predictions (i.e., those used in Figs. 3 and 4). The molecular differences that we establish between coral and algal DOM here with untargeted metabolomics are thus consistent with and extend a body of previous work showing how these two groups of benthic primary producers differentially influence water column biogeochemistry.

Studies observing microbial dynamics on reefs have documented patterns consistent with coral- and algal-dominated reefs exhibiting fundamentally different community structure and metabolism driven in part by differences in energetic content of DOM sources. When benthic community structure is changed on reefs, particularly when environmental conditions select for fleshy macro- and turf algae over calcifying organisms, both habitat loss and biogeochemical shifts lead to ecosystem degradation (51, 52). For over a decade, our research group has observed distinct characteristics of microbial community oxygen consumption and carbon demand when enriched with coral or algal exudates: in both field and shore-based mesocosm measurements, we have observed that coral exudates engendered microbial populations that grew slower, consumed less oxygen, and exhibited lower carbon demand and higher growth efficiencies compared to algae exudates (3, 4, 17). These experimental observations are recapitulated across reef states of coral to algal dominance in which algal-dominated sites exhibit lower DOC standing stocks, higher microbial cell abundances, significantly greater cell biomasses, and altered community metabolic capacity (16, 20, 32, 52, 53). One pivotal question emerging from these observations is how an increased carrying capacity of microbes with greater carbon demand is supported by algal-dominated reef states, and we hypothesize that the molecular composition of exudates, particularly aspects that regulate microbial growth efficiencies such as energy and nutrient content, may provide key insight into the mechanisms supporting this phenomenon. Our results, based on what can be observed through our analytical window, show that the exometabolite features derived from fleshy algae generate a pool of chemically reduced substrates with higher energetic potential than reef-builder exometabolite features (i.e., those from corals and CCA; Fig. 3).

### Mechanisms by which DOM Composition Is Posited to Influence Microbial Energetics.

**Fig. 6.**
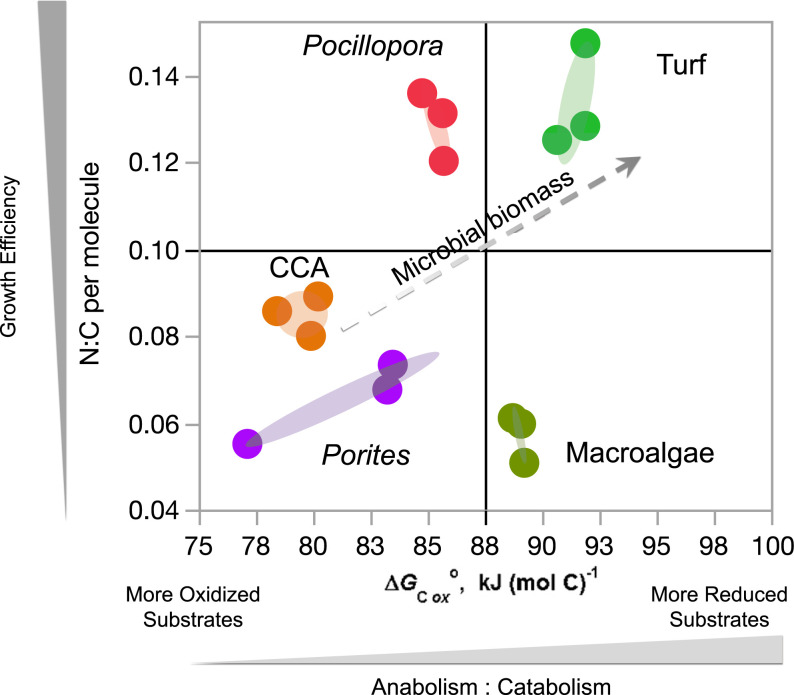
Conceptual illustration of the influence of benthic metabolites on the growth strategies of marine bacteria. Exometabolite samples are plotted according to weighted average exometabolite feature stoichiometric characteristics of Gibbs energy (Fig. 3) and N:C (Fig. 4 *A* and *D*) and annotated with hypothesized growth characteristics and biomass outcomes.

Fig. 6 depicts a working model that posits a direct linkage between fleshy algae-derived substrates and higher microbial biomass at degraded reef sites, potentially driven by the combination of reduced carbon oxidation states and elevated nutrient stoichiometry comprising the exometabolites of fleshy algae and particularly with algal turfs. The role of elemental stoichiometry and C oxidation states in determining the bioavailability and energetic content of DOM is inherently complex (18, 54, 55), though some chemical characteristics distinctly influence cellular metabolism. For instance, the energy potential gained during catabolism is greater in compounds with low C oxidation states (18). Cellular uptake of substrates with reduced C oxidation states has also been shown to increase proportional incorporation into the structural elements of microbial cells (i.e., anabolism) (55). Cells independently regulate rates of anabolism, catabolism, and orthogonal factors that alleviate resource limitation, including increased lability or nutrient content of DOM, increased growth efficiency, and decreased requirements for maintenance catabolism, such that both energy and nutrient content regulate different aspects of microbial biomass accrual and carbon utilization (56). We hypothesize that the production of more reduced, energy-rich compounds is one mechanism driving higher microbial growth rates on algal exudates; when combined with enriched labile sugars (not resolved by the LC-MS approach but described previously) (17), these exudates foster microbial community metabolic strategies that contribute to the observed microbialized states of high cellular biomass and rapid organic carbon removal (16). Our results also indicate significant enrichment of nutrients in the exometabolomes of corals (Fig. 4), and release from nutrient limitation is widely held to increase the growth efficiency of heterotrophic microbial communities (57, 58). Thus, we posit that the increased bacterial growth efficiencies observed in response to coral exudates (17) are facilitated primarily by the enhanced N and P availability measured in coral exometabolite features demonstrated here. Tradeoffs between microbial biomass yield and growth rate are thus fundamentally contingent on a deeper understanding of nutrient stoichiometry and structural characteristics of DOM (59), arguing that modern untargeted metabolomics has a central place in understanding microbial and ecosystem ecology.

### Reef Ecosystem Macronutrient-Recycling through the Exometabolite Pool.

Coral reefs have immense environmental significance because of their ability to support more species per unit area than any other marine system (13). Typically thriving in low-nutrient tropical waters, hermatypic corals have long been predicted to possess adaptive qualities to acquire, store, and recycle energy and nutrients efficiently (5, 43), but the mechanisms by which these processes occur have yet to be discovered. Our results show clearly that corals exude significant quantities of DOM enriched in phosphorus; these coral exometabolites had higher average P per molecule (Fig. 4*A*), a higher proportion of molecules containing P (Fig. 4*B*), and markedly higher concentrations of both total dissolved phosphate and DOP (*SI Appendix*, Figs. S3 and S11). The consistent release of organic phosphorus by corals has been observed previously (60) and has been identified as a crucial process to examine further in understanding reef ecosystem P dynamics (56). Together, these results suggest that corals are playing a key role in repackaging phosphorus in the reef to be subsequently remineralized by bacterioplankton. Further investigation into the chemical identity and residence time of DOP in the exometabolite pool may elucidate some of the ways in which reefs retain and recycle this critical macronutrient.

Coral reef nitrogen dynamics are far more complex, with multiple species of inorganic N playing different ecological roles on reefs (61), a significant role of animal nitrogen-cycling known to directly fertilize reef macrophytes (62), and the fundamental role of nitrogen exchange in regulating the Cnidaria–Symbiodiniaceae relationship in zooxanthellate corals (63). Our results reveal that some algae, namely turfing and crustose coralline varieties, contain significantly elevated quantities of organic nitrogen in their exometabolome (*SI Appendix*, Fig. S11*C*) through both elevated proportions of N-containing metabolites and exometabolite feature N content, while the macroalga *Dictyota* released DOM depleted in both N and P (Fig. 4 and *SI Appendix*, Fig. S11 *A* and *B*). The elevated nitrogen levels in the exometabolite features of Turf in our experiments may be due to a significant component of N-fixing cyanobacteria that form a variable portion of many turfing algal communities (64). Corals, particularly *Pocillopora*, also released DOM with elevated N levels (Fig. 4 and *SI Appendix*, Figs. S3 and S11), and both corals released significant quantities of what was detected as nitrate into the surrounding water. The relatively reduced quantity of organic matter and reduced nitrogen content of exometabolite features released by *Porites* relative to *Pocillopora* is consistent with other work contrasting these two coral genera with regard to environmental sensitivity and host regulation of the symbiont: *Porites* may more tightly control both absolute quantity and nutrient content of exudates through strong symbiont specificity (65, 66). Taken together, these results suggest that both macroalgal and coral-dominated reefs are likely to have a significant N-recycling dynamic between bacterioplankton and benthic primary producers, while declining coral cover may fundamentally alter P-recycling and availability in reefs. This is consistent with previous work as reef flats dominated by algal macrophytes can exhibit net drawdown in nitrate concentrations (consistent with *SI Appendix*, Fig. S3), while reef communities composed of mixed coral and algae often enrich the surrounding waters with nitrate and ammonia (39, 67–69). Our results have significant implications for the concept of nutrient-recycling in coral reefs generally, particularly emphasizing the potential for microbial repackaging and remineralization of benthic exudates as a retention mechanism. In particular, our results offer an alternative direction for the exploration of the role of sponges in Caribbean reefs in processing DOM into particulate organic matter as a mechanism for nutrient retention and food web recycling (70, 71). Recent work has shown the degree to which nutrients, particularly nitrogen, are remineralized or repackaged by sponges (72), and the contrasting role of sponges in reefs with strong coral or algal benthic biomass may be reflected in their differential processing of these two types of organic exudates (14, 73).

### Conclusions and Future Directions.

Assigning chemical information to the exudates of dominant reef benthic primary producers is a first and necessary step toward understanding the complex molecular matrix of the coral reef ecosystem (e.g., refs. 15, 32). Our study provides key insight into connections between benthic primary producers, DOM, and nutrient-cycling. The difference in nominal carbon oxidation state and macronutrient content of benthic producer exudate offers a stoichiometric underpinning to previously documented changes in microbial community structure observed in reef biomes undergoing phase shifts. Our characterization and identification of major classes of compounds distinct to corals, CCA, macroalgae, and turfing algae in reefs help explain the diverse molecular ecology of these ecosystems and provide a launching point for more targeted chemical analyses. While this study broadly characterizes the stoichiometry and chemical taxonomy of a fraction of benthic primary producer exudates, future studies need to investigate the microbial transformation and metabolism of these compounds to fully understand the role of exometabolites in key biogeochemical processes relevant to the resilience of coral reefs.

## Methods

Additional details on organismal collection, experimental logistics, biogeochemical analytical approaches, SPE of DOM, LC-MS/MS methods, calculations of NOSC, and data and code sharing are further detailed in *SI Appendix*.

### Mass Spectral Data Analysis.

MS/MS .raw data were converted to .mzXML in centroid mode using MSConvert (74). MS/MS spectra were analyzed with the Feature-Based Molecular Networking workflow (26) that annotates and connects related ion species in molecular networks within the GNPS web platform (22). MS1 feature extraction was performed with MZmine2 (version 2.31_corr_16.4) (29). Mass detection was performed in centroid mode with a minimum signal-height threshold of 1.0 × 10^5^ for MS1 level and 1 × 10^3^ for MS2 level. XICs were built with a minimum time span of 0.02 min, minimum peak height of 3.0 × 10^5^, and a relative mass tolerance of 10 ppm. Chromatographic deconvolution was performed with the baseline cutoff algorithm with a baseline level of 1 × 10^5^ and a minimum peak height of 3.0 × 10^5^ and peak duration range from 0.01 to 3.00 min. The maximum peak length was set to 2 min. Peak mass by charge (m/z) center calculation was set in Median mode, peak m/z range for MS2 scan pairing was set as 0.01 m/z, and RT range for MS2 scan pairing was set as 0.1 min. For isotope peak grouping, m/z and retention time tolerances were set to 10 ppm and 0.1 min, respectively, and a maximum charge of 4 was applied; the most intense peak was selected as a monoisotopic ion. For alignment of XICs between samples, we applied the following thresholds: 10-ppm mass tolerance with 75% weighting and 0.015-min retention time tolerance with 25% weighting. After alignment, the peak list-filtering option was used to select XICs that contained at least two isotope peaks and that occurred at least twice across all samples and had an MS2 scan. The aligned peak list was further filtered to remove as duplicates any XICs within 5-ppm mass windows and 0.1-min retention time windows. After filtering the aligned peak list, gaps in the feature matrix were filled with the original peak information using the multithreaded peak finder algorithm with a retention time tolerance of 0.1 min and 10-ppm mass tolerance. Consensus MS/MS of each feature were exported as .mgf files for molecular networking through the GNPS software infrastructure (https://gnps.ucsd.edu/ProteoSAFe/static/gnps-splash.jsp) (22). A spectral network was then created with a minimum cosine score of 0.7 and more than five matched peaks. Only the top 15 edges connecting one node were kept in the network. Consensus spectra were searched against the GNPS spectral library as well as National Institute for Standards and Technology 17 and Contaminants (22). Consensus spectra were additionally searched against the databases in analog mode using variable dereplication (22) with a maximum precursor delta mass of m/z 200. Library hits discussed in this article were inspected manually, and mirror plots of spectrum library matches are shown in *SI Appendix* (*SI Appendix*, Fig. S12). Molecular networks were visualized in Cytoscape 3.7 (75).

### Structural Prediction and Classification.

Features were annotated using spectral library matching via GNPS [including both library matches and analog matches via variable dereplication (22) and Network Annotation Propagation (31)]. Structural predictions (simplified molecular-input line-entry system [SMILES] or standard International Chemical Identifier [InChIs]) were then assigned a Direct Parent classification and chemical taxonomy from the ChemOnt chemical ontology via ClassyFire (24): ChemOnt provides a hierarchical structure for families of similar compound classes, allowing for groups of structurally related molecules to be compared across samples (e.g., Super Class = Lipids; Class = Fatty acyls; Subclass = Lineolic acids; and Direct Parent = Jasmonic acids). Features without subnetwork affiliations but with library or analog matches were given a consensus designation at the class level. Metabolite identifications in this work are generally putatively characterized and occasionally putatively analyzed at levels 2/3 of the Metabolomics Standards Initiative (76). Because individual exometabolite features clearly associated with specific experimental treatments (*SI Appendix*, Fig. S7), we tested the hypothesis that these associations would be found also at higher levels of chemical organization by evaluating the degree to which hierarchical chemical classes associated with specific experimental treatments (Fig. 5). To statistically assess congruency between exometabolite treatment association categories (*SI Appendix*, Fig. S7) and exometabolite structural classes assigned to annotated features or from network propagation methods, we compared the coefficients of determination from contingency analysis with χ^2^ *P* < 0.001. Out of the proportion of exometabolite features that were successfully networked (1,097 features or 66%), exometabolite subnetwork identity had the highest congruency with the experimental treatments (*R*^2^ = 0.83); higher-level ClassyFire classifications of those subnetworks by MolNetEnhancer were the next most congruent (*R*^2^ = 0.50). Of the ∼40% of exometabolite features matching library/analog identifiers, ClassyFire structural classifications showed similar congruency using only networked exometabolite features (471 or 43%; *R*^2^ = 0.55) or all exometabolite features (679 or 41%; *R*^2^ = 0.51). Because molecular subnetworks provided the highest congruency with ecological patterns, we chose to use spectral correlation networking in our final classification approaches (Fig. 5).

### Molecular Formula Assignment (SIRIUS, ZODIAC).

The tandem mass spectrometry data were annotated with the SIRIUS computational annotation tool (version 4.0.1 linux build 5) (25), running on a cluster computer (32 cores, 256 Gb of random access memory). De novo molecular formulas were computed with the SIRIUS module by matching the experimental and predicted isotopic patterns (77) and from fragmentation trees analysis of the fragment ions (78). Molecular formula prediction was refined with the ZODIAC module by using Gibbs sampling for fragmentation spectra that were not chimeric spectra or had a poor fragmentation (30). Parameters for SIRIUS and ZODIAC were set as follows: molecular formula candidates retained = 80, maximum precursor ion m/z computed = 700, profile (orbitrap), m/z maximum deviation (10 ppm), all adducts were considered (–auto-charge), adduct annotation from MZmine was trusted (–trust-ion-prediction); and ZODIAC threshold filter (0.995). We excluded 635 of 1,667 exometabolite features from all stoichiometric analyses for which we could not confidently assign a high-quality molecular formula (ZODIAC score >0.98, nonchimeric, and at least one fragmentation tree must explain >4 fragments and >80% spectral intensity), retaining for molecular formula analyses a subset of 1,032 exometabolite features (Figs. 3 and 4 and *SI Appendix*, Figs. S9–S11).

### NOSC and Gibbs Energies of Oxidation Half Reactions.

The processes of photosynthesis and respiration occur through oxidation–reduction reactions required for the formation (anabolism) and degradation (catabolism) of biomolecules, and the oxidation state of carbon is a primary determinant of the energy content of macromolecules (79). Photosynthesis reduces CO_2_ to energy-rich sugars with an average C oxidation state of 0 and subsequent metabolic reactions further reduce the oxidation state to negative values (e.g., proteins or lipids with C oxidation states between 0 and −4) (80). Catalysis of these organic substrates through electron transport and oxidative respiration cycles carbon between redox states and finally back to CO_2_ (C oxidation state of +4), with the free energy released dictated in part by the stoichiometry and structure of the compound. For each organismal sample, we calculated a NOSC weighted by the carbon content (the product of feature abundance and formula carbon count) of each feature statistically enriched in the exometabolite pool for that organism (*SI Appendix*, Fig. S5) and with a valid molecular formula (*n* = 1,032 features total). LaRowe and Van Cappellen (38) estimated the energetic potential of organic compounds based on major element (C, H, N, O, P, or S) ratios by relating the Gibbs energies of half reactions required for the complete mineralization of the compounds to their average NOSC as detailed in *SI Appendix*.

### Statistical Analyses.

All MS1 features with MS/MS spectra were compiled as raw peak areas in each sample. Method blanks (n = 8; LC-MS grade water processed in parallel with samples, including pumping, filtration, PPL binding, washing, and elution) were used to identify and remove features suspected to be procedural artifacts before data analysis: features were flagged as “background features” and removed if the mean peak area across all field and mesocosm samples (n = 250) was less than twice the maximum peak area across all blanks (n = 8). We defined “transient features” as features below a threshold peak area in less than three of the 42 samples in this experiment and were removed before analysis; threshold peak area was defined as the smallest feature peak area before gap-filling (2 × 10^5^: double the defined noise threshold). We differentiated features as “ambient features” or “exudate features” as those with a mean fold change in any exudation incubation treatment from the starting reef water of less than or greater than 2, respectively (defined as exceeding a threshold of 1 in the log2 ratio of mean final peak area in each treatment to mean starting peak area). Exudate features were defined as statistically significant “exometabolite features” exuded if their relative abundance was enriched in at least one of the five organismal incubations relative to the Control aquaria at the end of either daytime or nighttime incubations (ANOVA with Dunnett’s post hoc FDR-adjusted *P* < 0.05). The remaining exudate features were classified as “benthic exudates” if they were twice the mean peak area of ambient seawater in one or more organismal incubation treatments or “planktonic exudates” if twice the mean peak area of ambient seawater in either Day or Night Controls; a small proportion of exudate features were flagged as likely artifacts of incubation if they were twice the mean peak area of ambient seawater in both Day and Night Controls and were not analyzed further (*SI Appendix*, Fig. S6).

## Supplementary Material

Supplementary File

## Data Availability

All MS data are publicly accessioned in the Mass Spectrometry Interactive Virtual Environment (MassIVE) repository (https://massive.ucsd.edu/ProteoSAFe/static/massive.jsp) with accession number MSV000082083. Raw data frames, statistical analyses, and R code are publicly accessioned from GitHub via Zenodo (DOI: 10.5281/zenodo.3959803). Molecular networking and spectral library comparisons are deposited in GNPS as follows: https://gnps.ucsd.edu/ProteoSAFe/status.jsp?task=f93cb26f95d94a0c84757dd0eb0bb408; https://gnps.ucsd.edu/ProteoSAFe/status.jsp?task=d7373e2add2a4e72a871a60c96fbbbc3.
