# Supplementary material for: Distinguishing the molecular diversity, nutrient content, and energetic potential of exometabolomes produced by macroalgae and reef-building corals
Source: Proc Natl Acad Sci U S A. 2022 Jan 31;119(5):e2110283119. doi: 10.1073/pnas.2110283119 (PMC8812564; doi:10.1073/pnas.2110283119)
Supplement: Supplementary File [file pnas.2110283119.sapp.pdf]

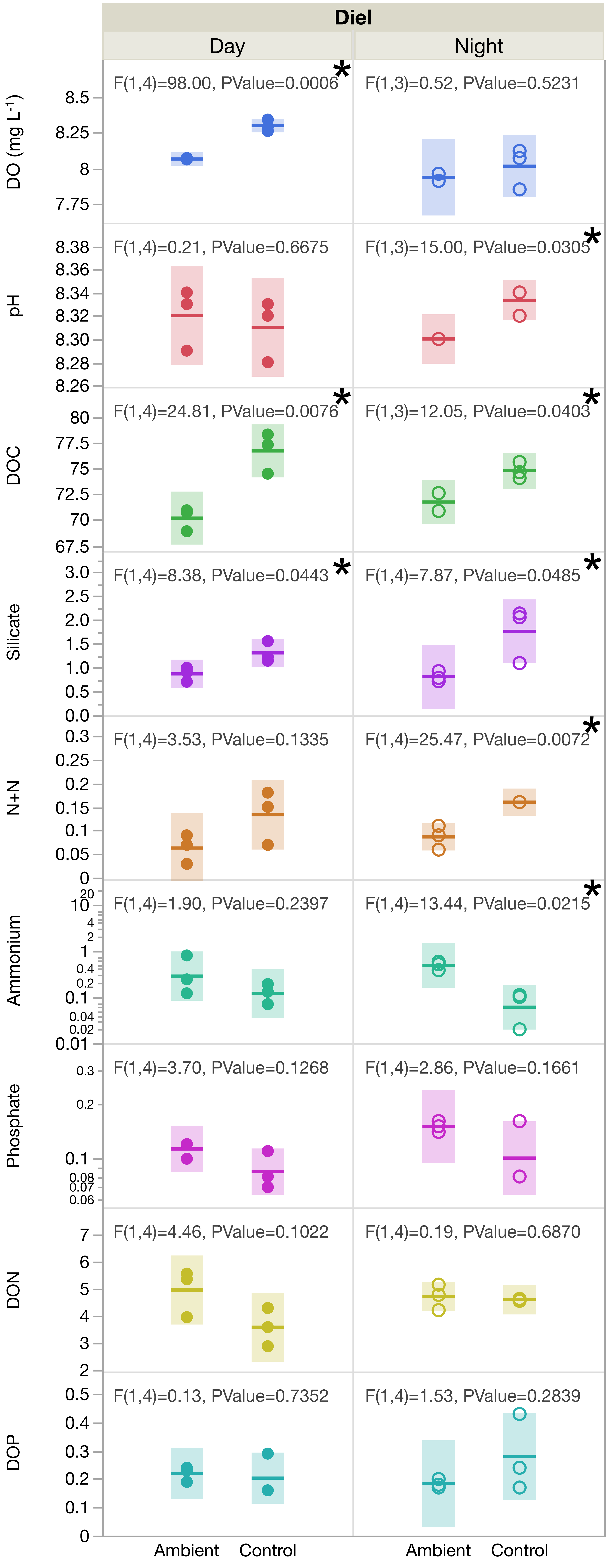

Figure S1. Effects of seawater control incubations on all biogeochemical parameters. Parameters include DO, pH, DOC, DON, DOP, N+N (nitrate plus nitrite), NH4, PO4 and SiOX; all units in µM except DO as noted. We used 2-way ANOVA to evaluate differences between starting (Ambient) and ending seawater controls (Water) in the day and night. No interaction terms were significant (p > 0.05) and only oxygen differed significantly between day and night samples (p_{diel} = 0.0043) with higher daytime means (8.06) than nighttime means (7.93) in the ambient starting water. Statistics for t-tests are shown above each comparison. Oxygen increased in the daytime incubation control. Both DOC and SiOx increased slightly in the seawater controls over the incubation periods of both daytime and nighttime samples; in the nighttime samples pH and N+N increased while Ammonium decreased. There was no change in PO4, DOP or DON in controls.

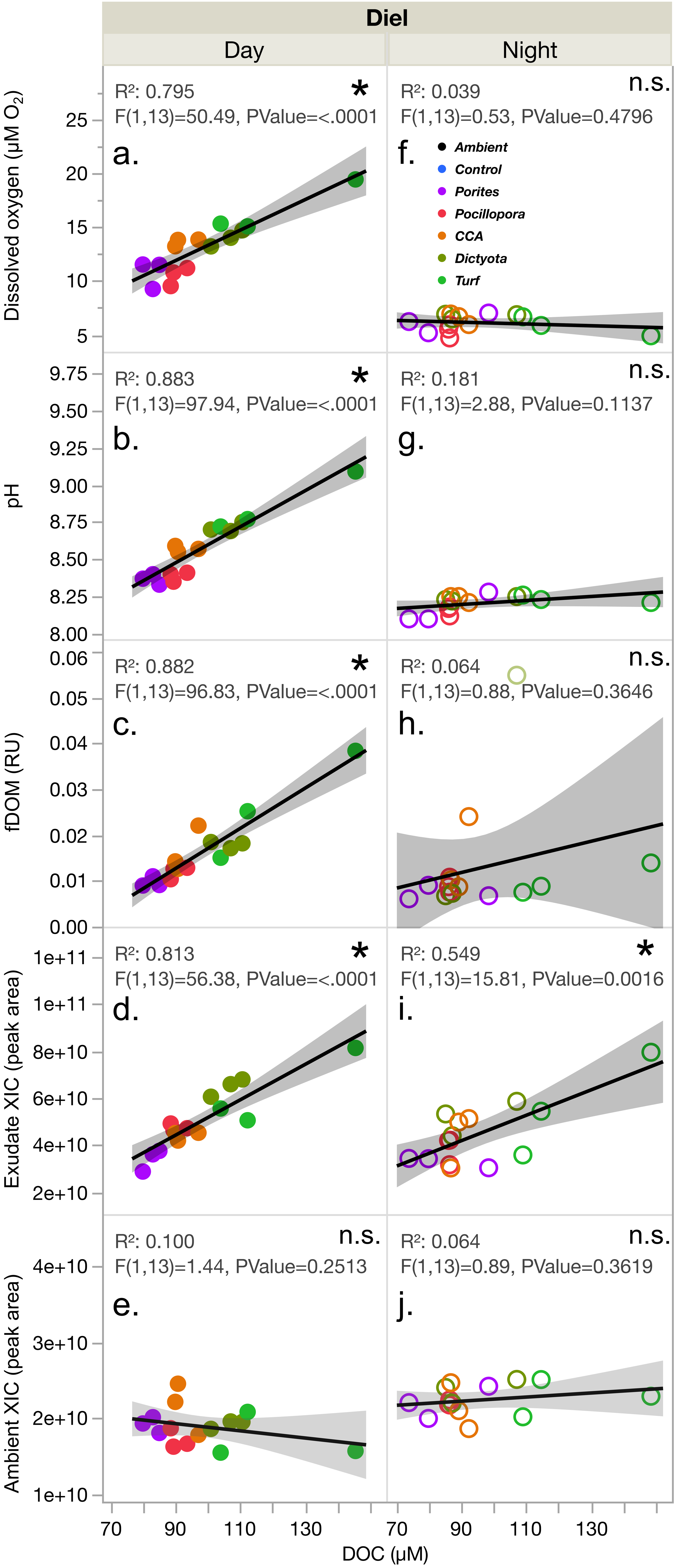

Figure S2: Covariation of DOC produced with oxygen, pH and bulk DOM compositional change across daytime and nighttime treatments. Model statistics are noted at the top of each panel and flagged as significant (*) or nonsignificant (n.s.) at alpha of 0.05. Note that DOC production tracks oxygen evolution (a), pH increase (b) and production of both humic fDOM (c) and exudate metabolite features (d) in daytime incubations, but not at night (f, g, h) except nighttime exudate feature production is related to DOC (i). Ambient feature production does not correlate with DOC release in day or night incubations (e, j)

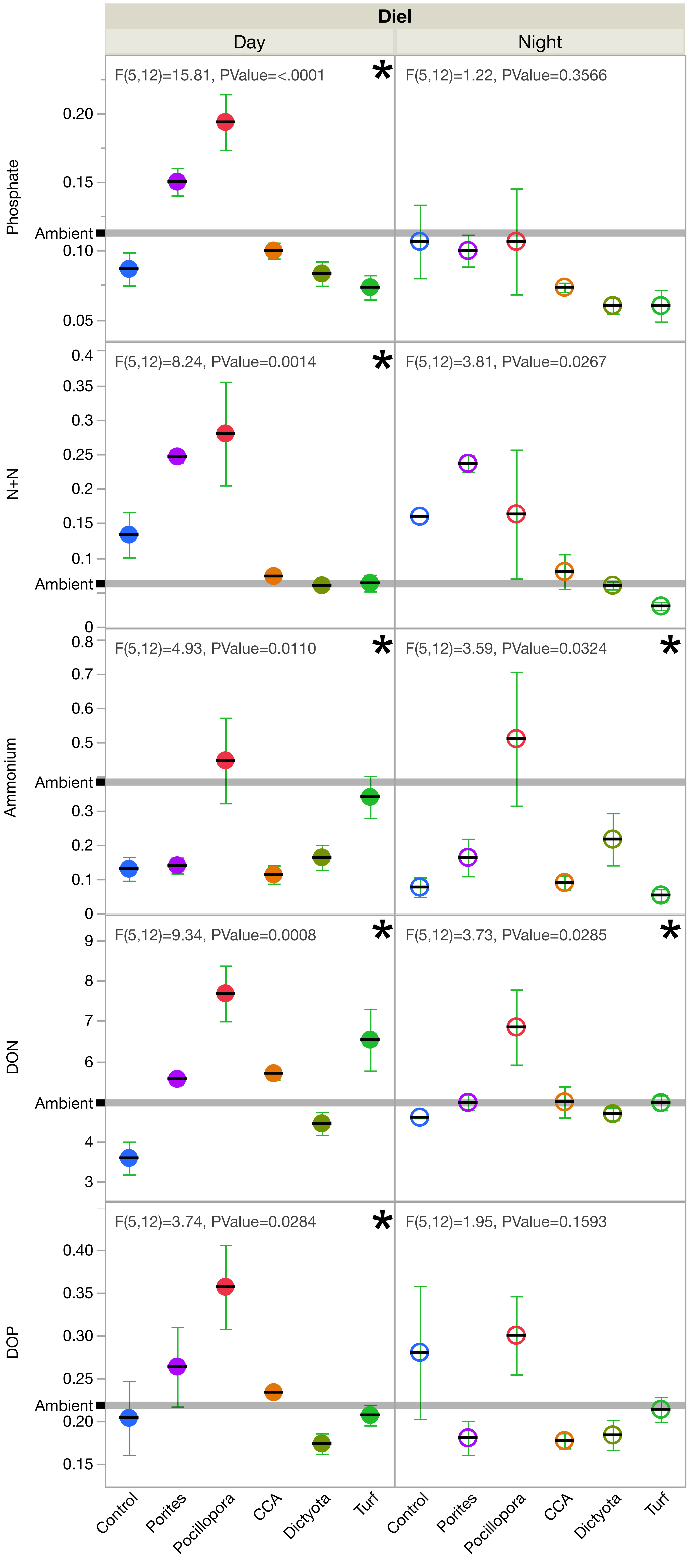

Figure S3. Effects of benthic producers on macronutrient parameters. All concentrations are in micromolar; N+N refers to nitrate+nitrate. Asterisks designate significant enrichment relative to the seawater controls using ANOVA with Dunnet's *post hoc* tests. Ambient lines represent the mean starting seawater concentrations measured at the start of the experiments before incubation.

- ***Ambient***
- ***Control***
- ***Porites***
- ***Pocillopora***
- ***CCA***
- ***Dictyota***
- ***Turf***
- Day
- Night

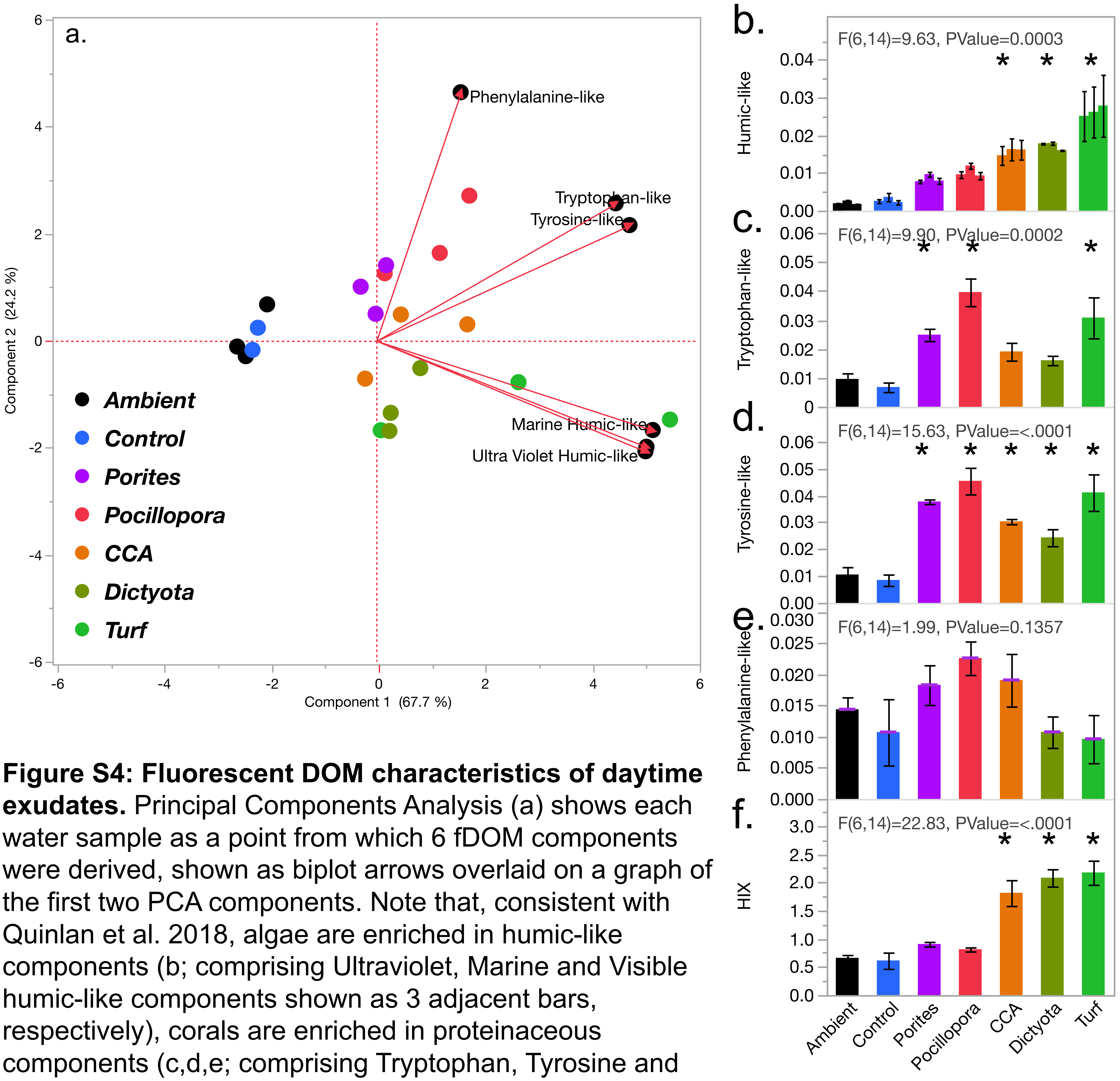

Figure S4: Fluorescent DOM characteristics of daytime exudates. Principal Components Analysis (a) shows each water sample as a point from which 6 fDOM components were derived, shown as biplot arrows overlaid on a graph of the first two PCA components. Note that, consistent with Quinlan et al. 2018, algae are enriched in humic-like components (b; comprising Ultraviolet, Marine and Visible humic-like components shown as 3 adjacent bars, respectively), corals are enriched in proteinaceous components (c,d,e; comprising Tryptophan, Tyrosine and Phenylalanine-like components, the latter nonsignificant). HIX stands for the widely used fDOM humification index. Statistically significant parameters in 1-way ANOVA tests are shown (b-f) with means and standard error whiskers at right as well as asterisks denoting treatments significantly enriched relative to the control with Dunnet's *post hoc* tests.

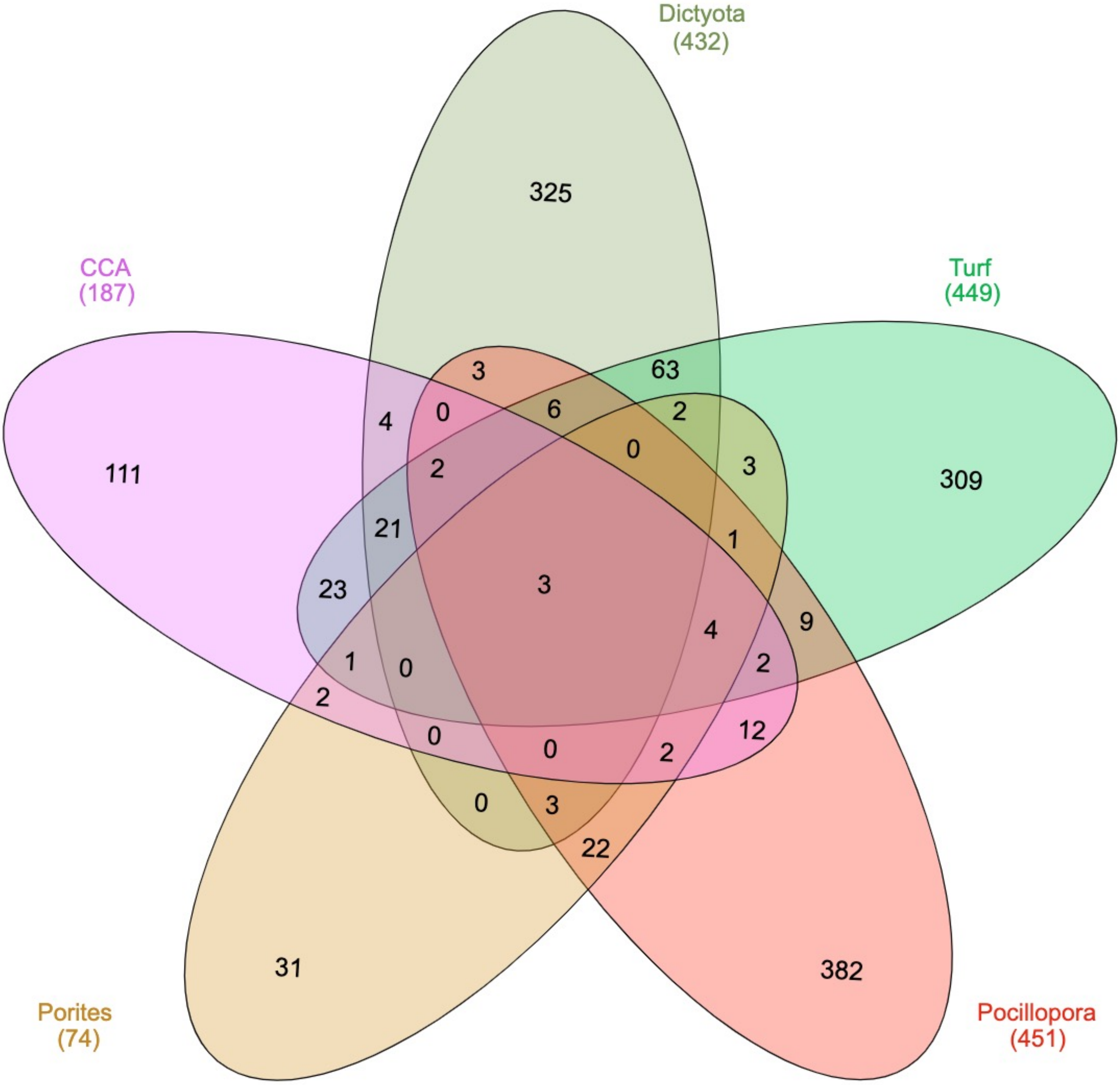

Figure S5. Venn diagram of the number of shared exometabolite features among daytime benthic producer treatments. Note that of the 1346 features significantly enriched in at least one of the daytime treatments, the vast majority (86%; 1158) of the features are uniquely significantly enriched in only one of the five treatments, and an additional 10% (141) in two of the five.

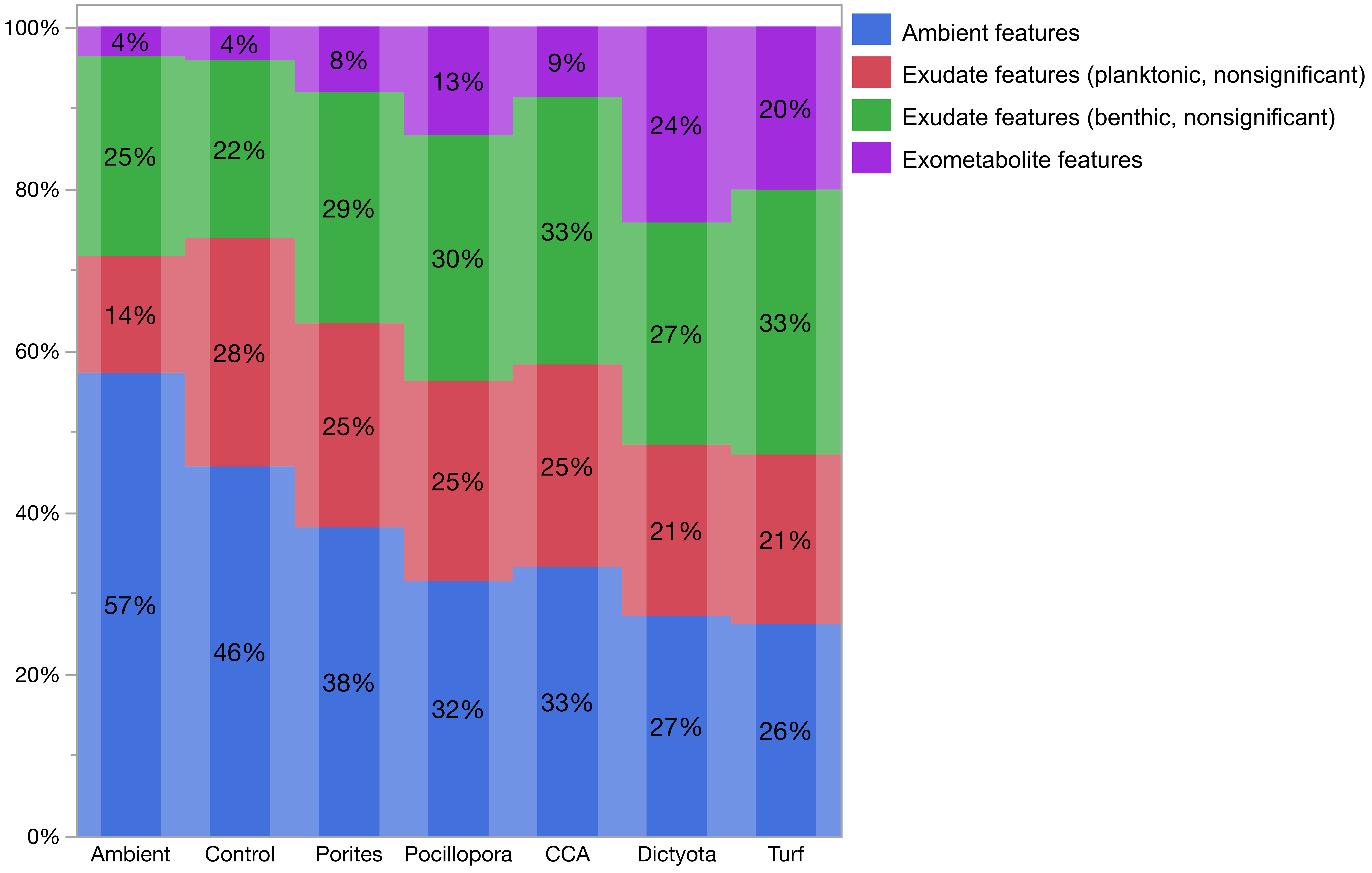

Figure S6. Mean proportion of spectral peak area (XIC, MS1) allocated to feature types in different treatment categories. Ambient features are those statistically enriched in Ambient samples and never exceeding twice the reef water values as defined in the methods. Exudate features are twice the reef water values in at least one incubation treatment as defined in the methods; those enriched in the Control are considered planktonic and those enriched only in benthic organismal treatments are considered benthic. Exometabolite features are statistically significantly enriched in one of the five benthic organismal treatments relative to the Control as defined in the methods. Due to the stringent pairwise false discovery rate controls applied to the statistical differentiation of exometabolite features (1,667 released consistently by a benthic producer) from exudate features (8,936 enriched above the ambient reef water) the vast majority of the latter (81%, or 7,269) were classified as those enriched in any of the five benthic macroorganisms (4,846 benthic exudate features twice the mean peak area of ambient seawater in one or more organismal incubation treatments) as well as those potentially produced by the remaining planktonic community after 0.2 µm pre-filtration (2,047 planktonic exudate features twice the mean peak area of ambient seawater in either Day or Night Controls) or likely artifacts of incubation (376 features that increased in both Day and Night Controls over 8 h).

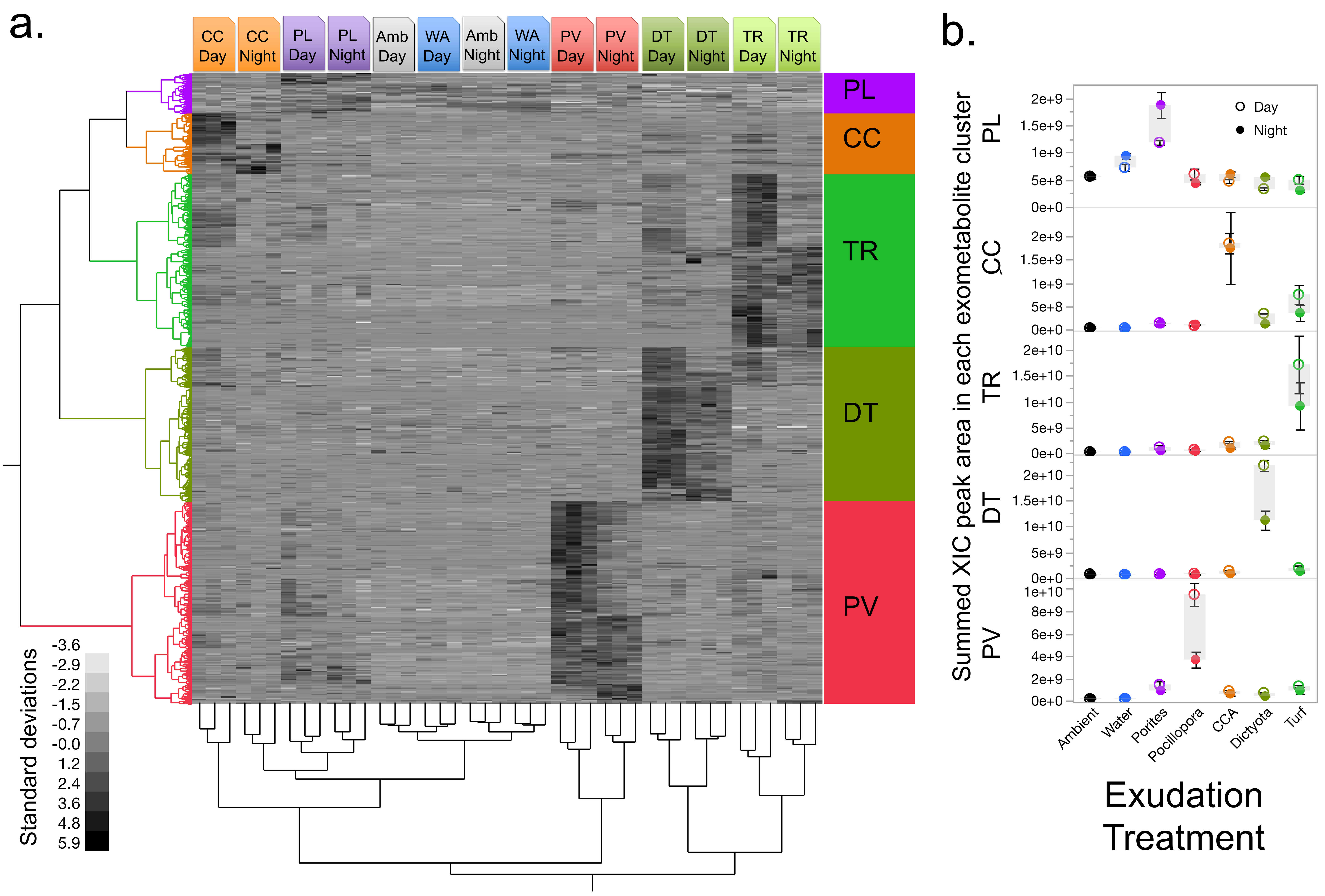

Figure S7. Clustering of exometabolite features. Relative abundances of exometabolite features (n=1667) were internally standardized using z-scores (heat map) and hierarchically clustered using Ward's Minimum Variance method (a). Five feature clusters were resolved (dendrogram colors at left and 2 letter codes at right: *Porites lobata* = PL, CCA = CC, Turf = TR, *Dictyota* = DT, *Pocillopora verrucosa* = PV), each enriched most prominently in one of the five benthic producer incubation treatments (top), with replicate treatments clustering together (bottom, also Figure 2). Note that within each exometabolite type features are enriched either primarily in the daytime (upper subcluster) or both day and night (lower subcluster). Panel (b) shows the raw summed MS1 XIC peak area attributed to each exometabolite feature cluster (2 letter codes from a) in each experiment day and night, emphasizing the specificity of these metabolite clusters both in quality (a) and in quantity (b).

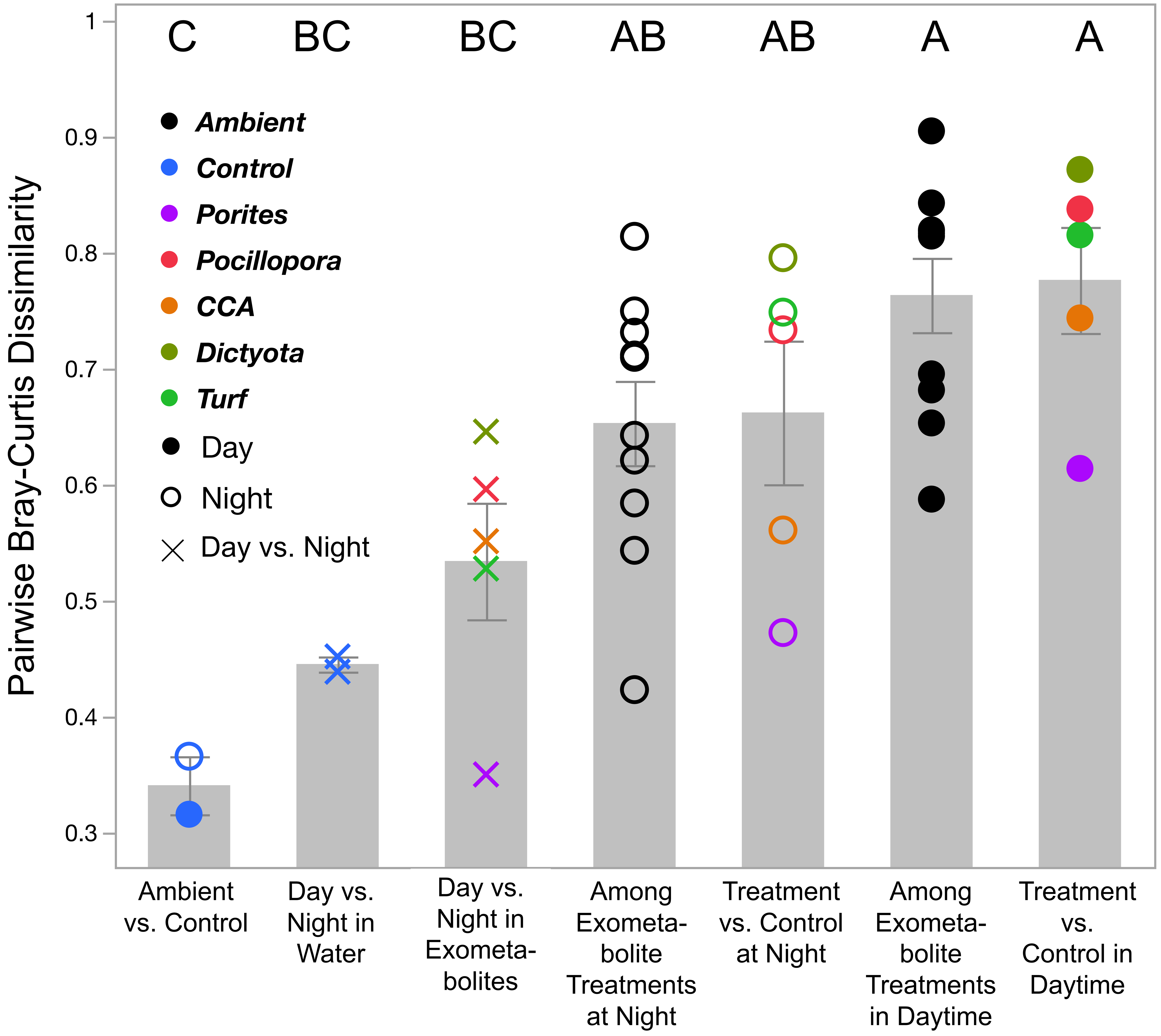

Figure S8. Pairwise Bray-Curtis dissimilarities in exometabolite relative abundance among samples from different treatments. The categories across the bottom group the pairwise dissimilarities (points) according to the type of comparison, and means (grey bars) with the same letter are not significantly different at alpha = 0.05 (ANOVA with Tukey *post hoc* test). The starting (Ambient) and ending (Control) water exhibited the smallest variation in exometabolome composition (mean bray-curtis dissimilarity 0.34); the mean diel difference within water treatments (0.44) or within benthic producer treatments (0.53) was significantly less than the differences between exometabolomes during the day (0.76) or the differences between exometabolomes and water controls during the day (0.78), with nighttime dissimilarities intermediate (0.66).

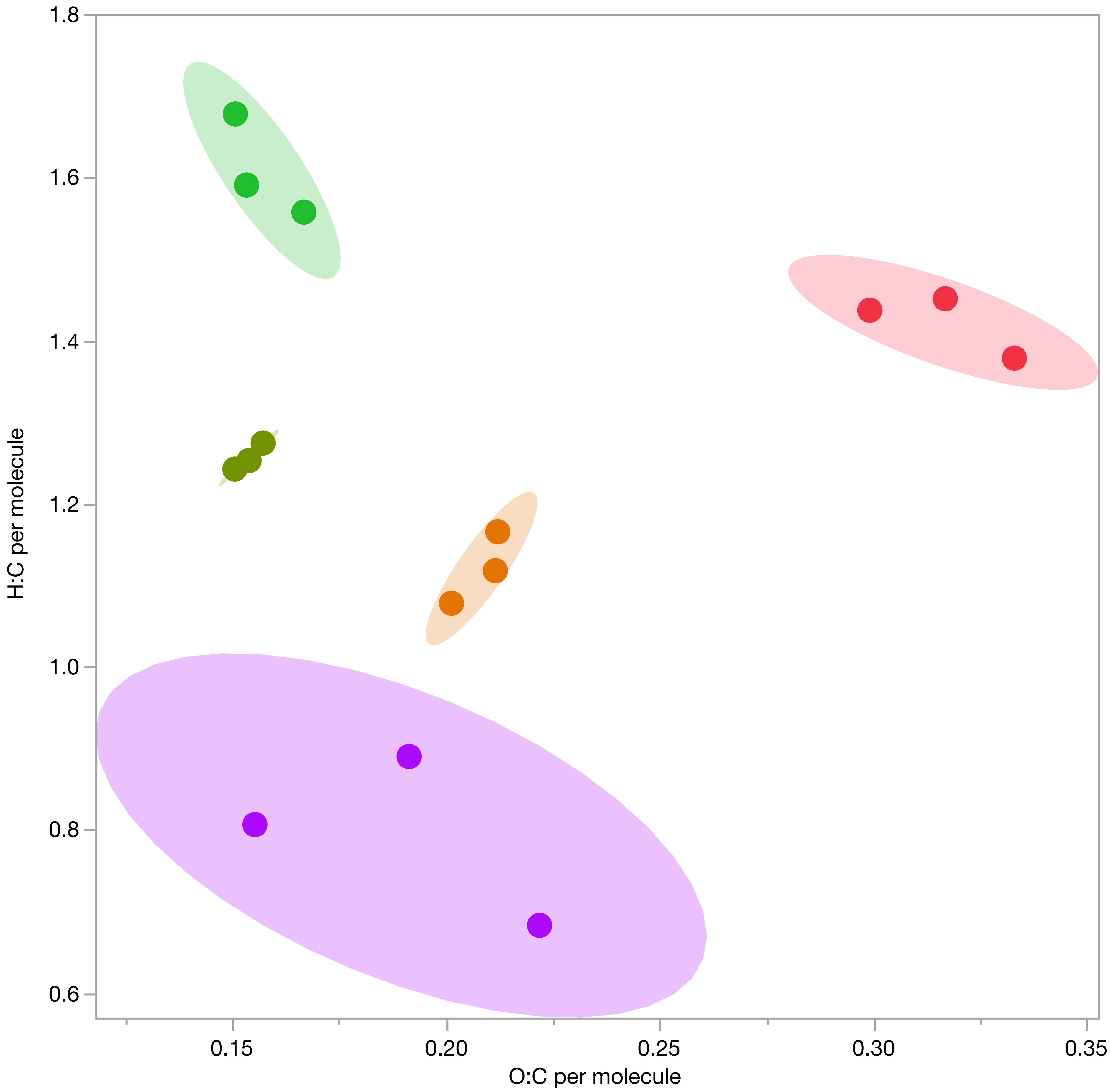

Figure S9. Weighted mean daytime exometabolite oxygen to carbon ratio (O:C) and hydrogen to carbon ration (H:C) in Van Krevelen space. Each point is one replicate incubation exometabolite sample.

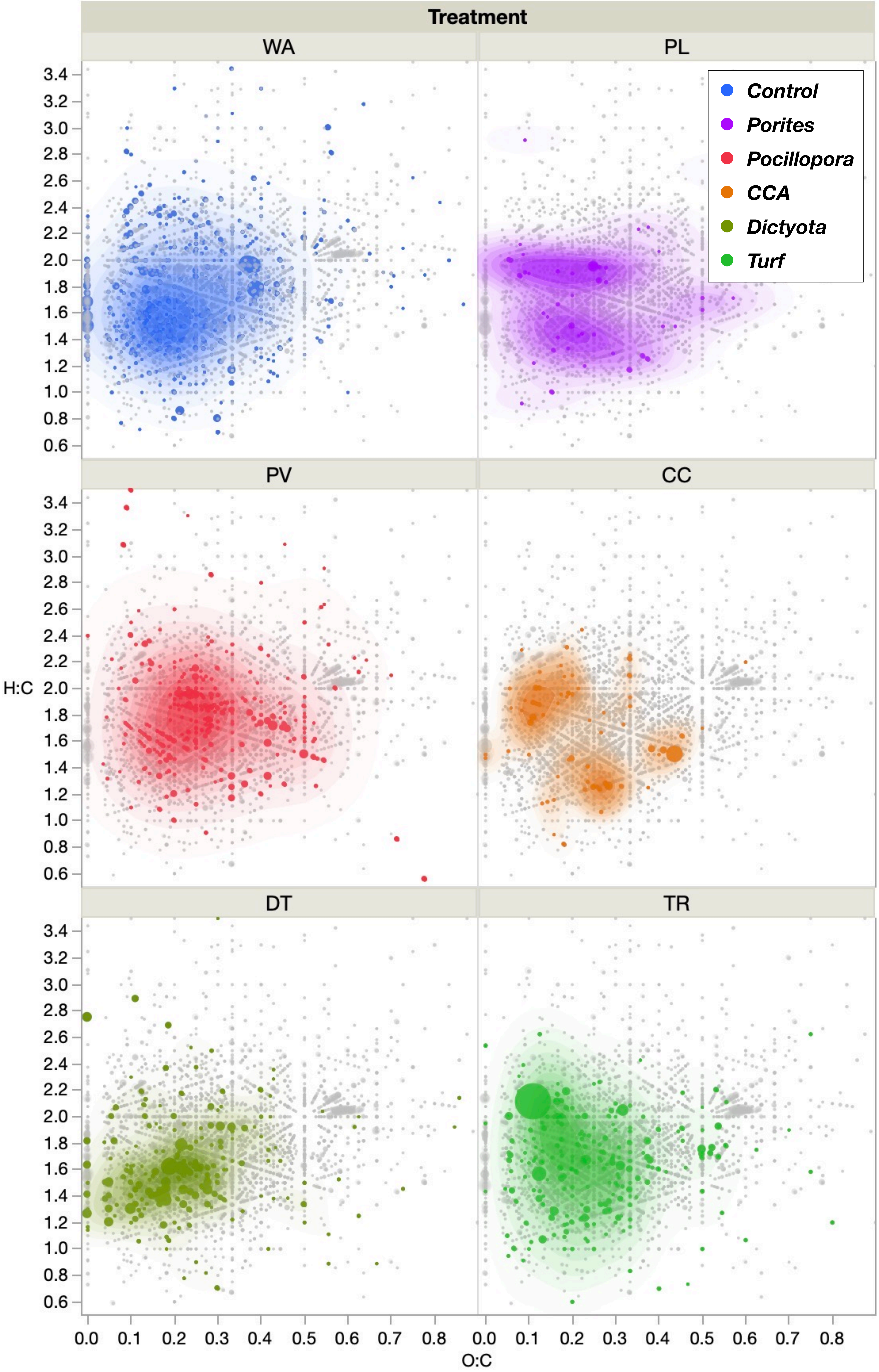

Figure S10. Distribution of all exometabolites in Van Krevelen space. Grey points are ambient metabolites with colored points and nonparametric density contours overlaid corresponding to features enriched in specific exometabolite samples (Two Letter Treatment Codes defined in Figure S7; *Porites lobata* = PL, CCA = CC, Turf = TR, *Dictyota* = DT, *Pocillopora verrucosa* = PV, Water Control = WA). Points are sized according to average XIC in each treatment.

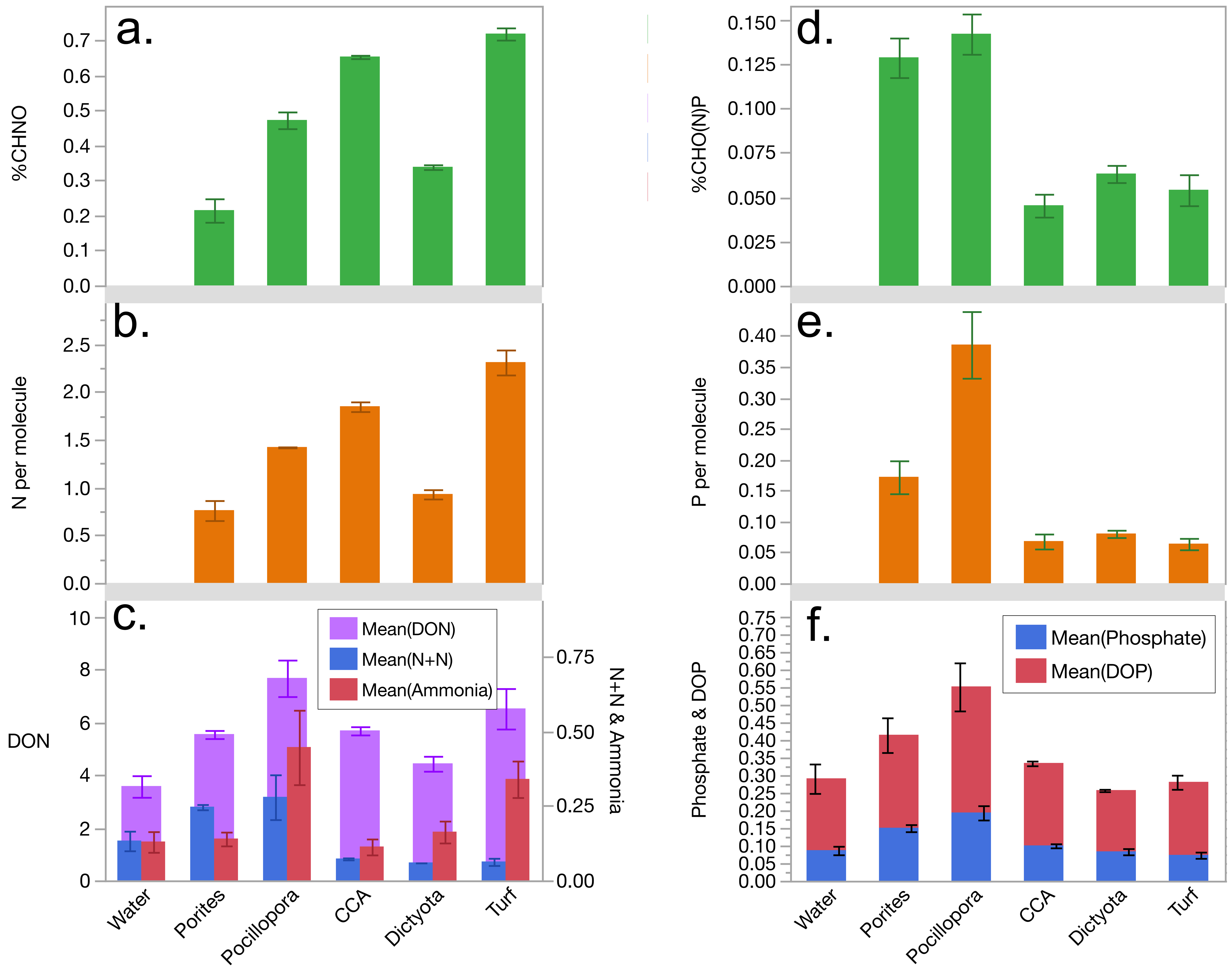

Figure S11. Contrasting elemental stoichiometry of daytime exometabolites with seawater N and P chemistry. (a and d) show the proportion of exometabolite features with stoichiometries containing N or P, (b and e) show weighted mean (+/- s.e.m.) N and P per molecule in exometabolite features. (c and f) show mean concentrations (micromolar) of dissolved organic N and P as well as dissolved inorganic N and P species in daytime exudate pools.

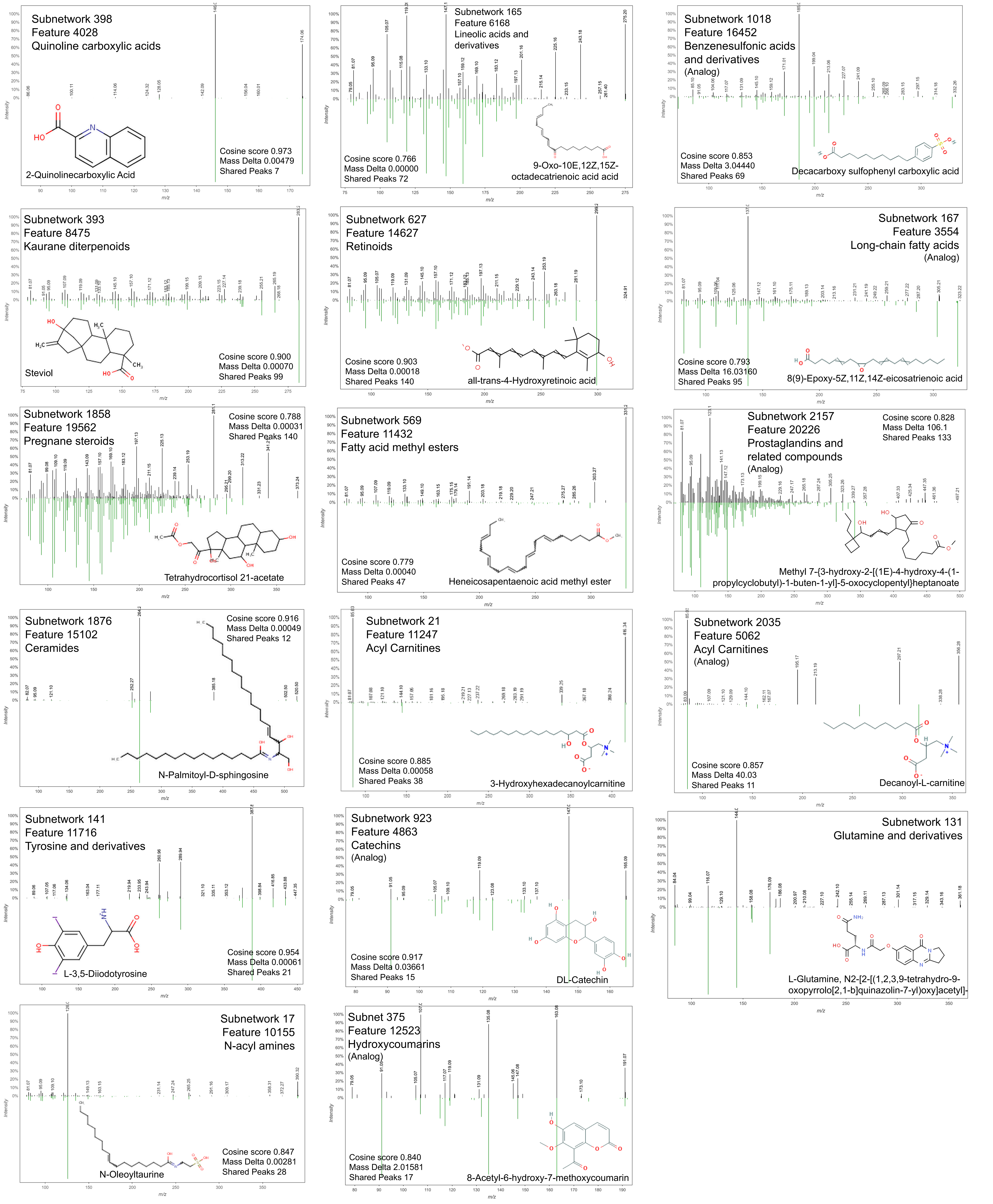

Figure S12. Structural annotations of MS2 fragmentation spectra curated as high quality matches to known compounds from public databases. Mass deltas above 0.01 m/z are from variable dereplication and labeled (Analog). Cosine Score provides spectral similarity to the database match among the Shared Peaks. Structures and names of mirrored spectra are provided.

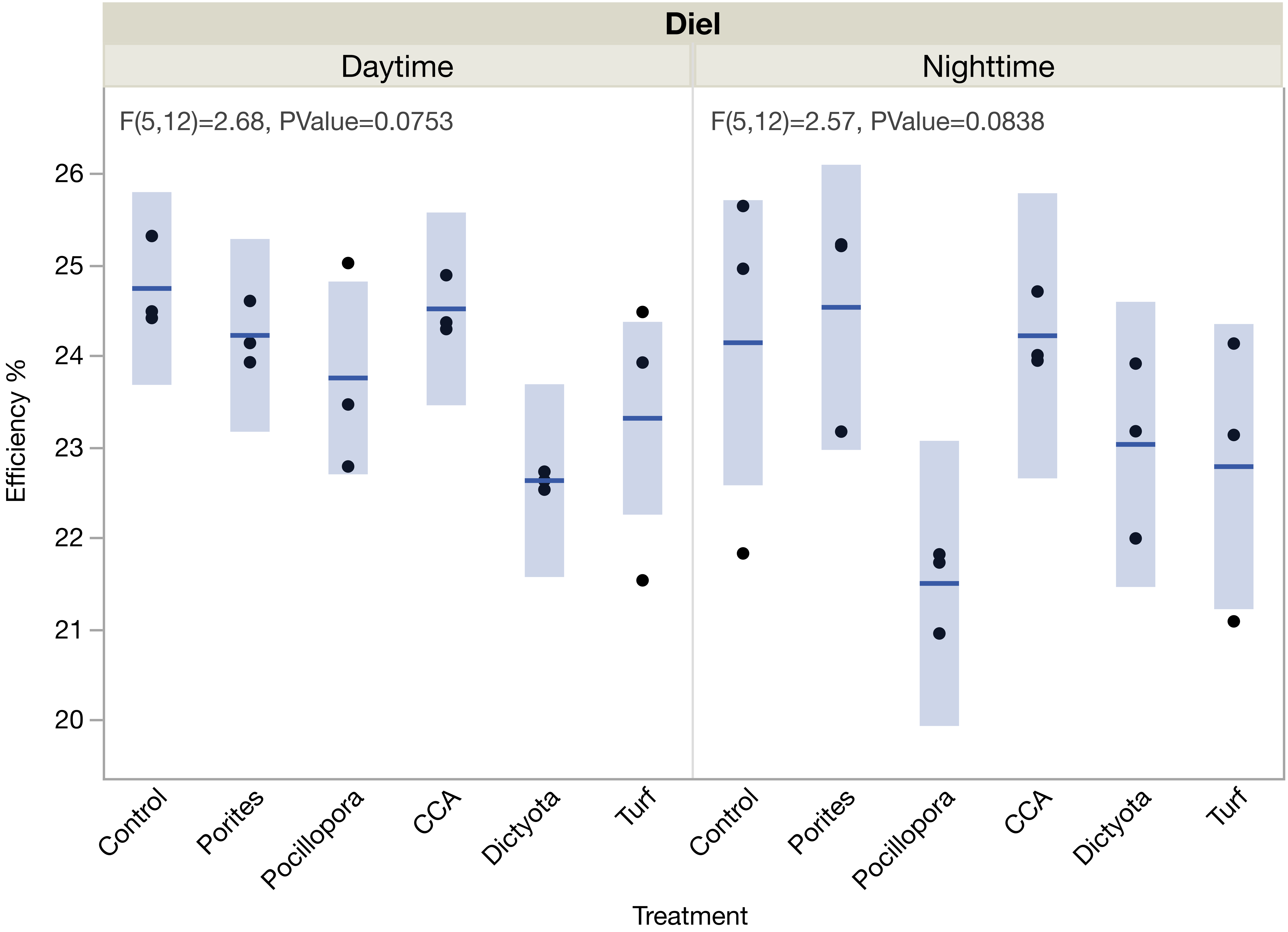

Figure S13. Extraction efficiencies of DOC from PPL. All efficiencies were measured on endpoint samples. ANOVA statistic at top indicates no significant difference among treatment mean efficiencies in either Daytime or Nighttime exudation experiments ($p > 0.05$).

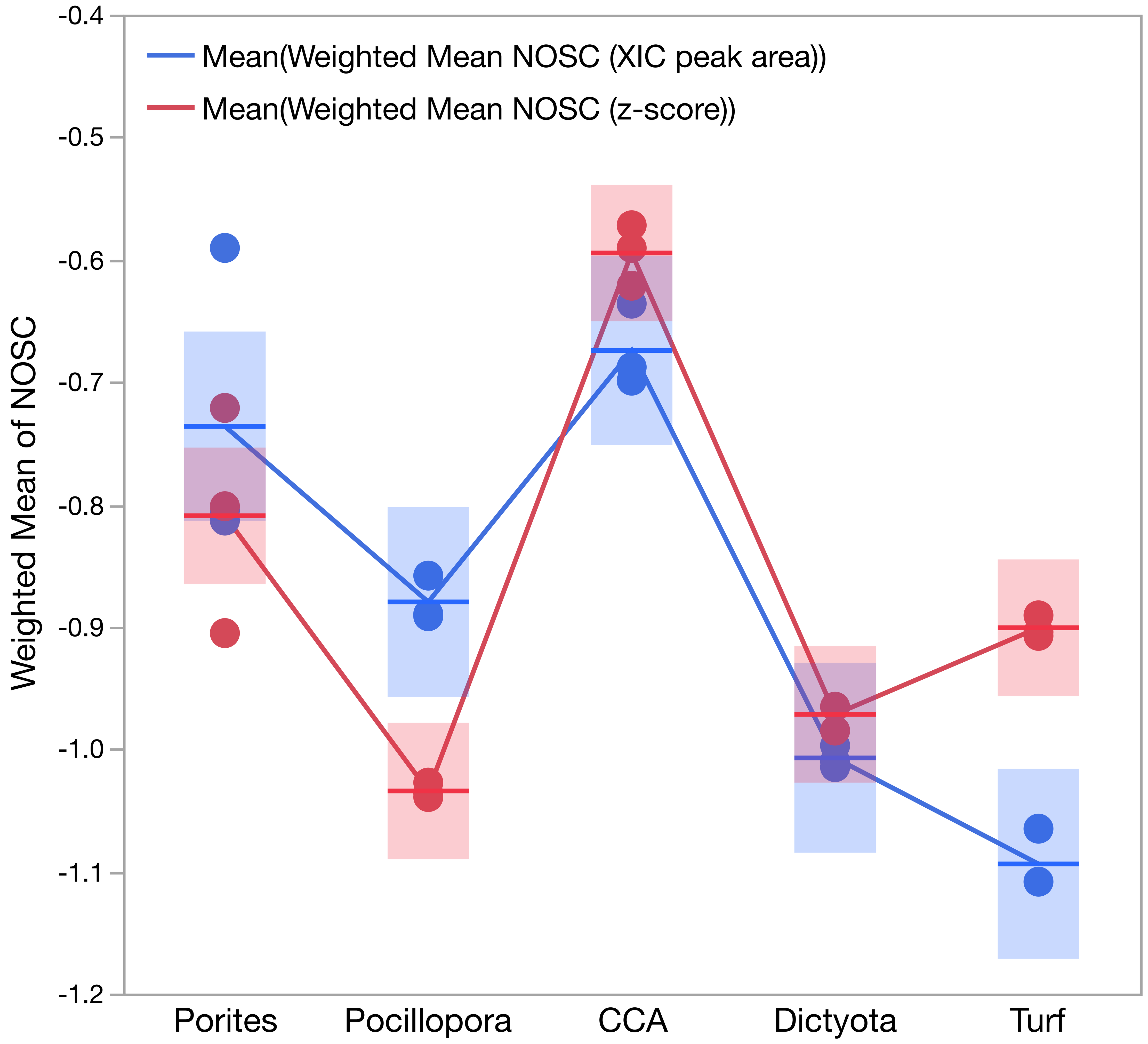

Figure S14. Comparison of the effect of standardized vs. raw peak area summation on calculation of weighted mean NOSC. The blue values match those presented in Figure 3 while the red first convert all peak areas to standard scores (using the z-scoring approach) to eliminate the potential bias of differential ionization of exometabolite features.

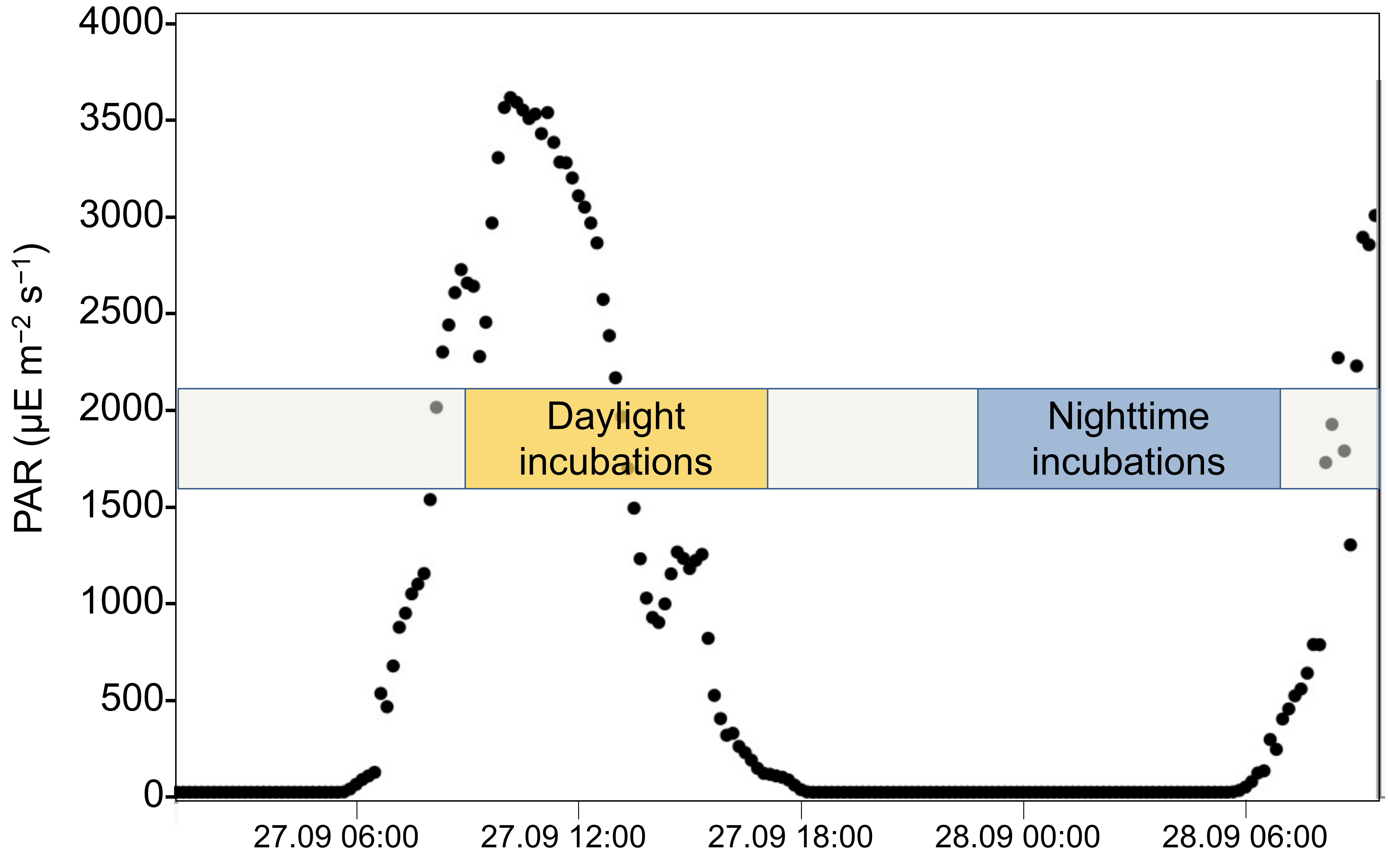

Figure S15. Light availability in incubation tanks during the period of day and night incubations. Light intensities were assessed as photosynthetically active radiation (PAR) using an Odyssey® Waterproof Photosynthetic Active Radiation Logger.

Supplementary Methods:

Organismal collection and handling. All specimens were collected in triplicate from water depths of 2.0–3.5 m at backreef locations (17°28'55"S, 149°50'43"W) five days prior to the experiment (22-23 September) in the backreef habitat between the north facing Paopao and Ōpūnohu bays and transferred in coolers without air exposure to flowing seawater cultivation tanks with ambient temperature and light within 1 hour of collection. Specimen surface areas did not differ significantly among treatments and averaged 124 cm^2. Exudate generation was conducted 27-28 September. Incubations were conducted separately over a daytime period (0900 – 1700) and, after a water exchange, over a subsequent nighttime period (2300 – 0700) to assess differences in daytime and nighttime organic matter release. Each specimen was placed into one 1.5 L polycarbonate incubation container (acid washed, leached for 24h in ambient flowing seawater, then rinsed with acid and deionized water) and incubated with a fitted lid and no agitation or bubbling filled with freshly collected "sterile" reef water (filtered through pre-flushed 142 mm polyethersulfone 0.2 µm pore size filters). The incubation containers were randomly placed on a shaded flowing water table to standardize light and temperature (temperature ranged from 25.9 to 26.8 degree C, light distributions are shown in Figure S15). To assess initial water parameters, triplicate samples of 1.2 L filtered reef water (seawater Control T0; technical replicates) were immediately processed and at the end of each incubation period, 1.2 L water was collected from each container (triplicate biological replicates) and processed for a suite of measurements of water chemistry.

Biogeochemical measurements: For measuring microbial abundance a 2 mL whole water sample was collected in a polyethylene screw top vial and immediately fixed to 0.5% paraformaldehyde and stored at -80°C until analysis by flow cytometry following Nelson et al. (2015). Measurements of pH and DO at the start and end of each incubation chamber were taken with a Hach HQ40D multiparameter instrument with Intellical probes (DO: precision 0.01 $mg.L^{-1}$, accuracy ±0.05%; pH: precision: 0.001 pH, accuracy: ±0.02 pH). At the end of each incubation the seawater was filtered through a 0.22 µm polyethersulfone filter (Millipore Sterivex). Samples for inorganic and organic nutrient analysis were collected in 50 mL polypropylene tubes and frozen -20 °C until analysis on a Seal AA3 Segmented Flow Injection Autoanalyzer at the

University of Hawai'i SOEST Laboratory for Analytical Biogeochemistry; nitrate+nitrate and silicate (Grashoff et al, 1983), ammonium (Kérouel and Aminot 1997), phosphate (Murphy and Riley 1962), and total dissolved N and total dissolved P were measured (the latter two with separate injections with UV and alkaline or acid persulfate in-line oxidation, respectively) and dissolved organic N and P (DON, DOP) were calculated as the difference between total and inorganic analytes. After flushing a 0.22 Sterivex filter with 100ml sample water samples for dissolved organic carbon (DOC) and fluorescent dissolved organic matter (fDOM) analysis were collected in precombusted 30ml glass vials with Teflon septa; DOC samples were acidified with trace metal grade HCl (J.T. Baker, USA) to a pH of 2 and stored at room temperature until analysis and fDOM samples were refrigerated. Analysis of DOC was done on a Shimadzu TOC-V at the UCSB DOM Analytical Laboratory following Carlson et al. (2010). Analysis of fDOM was done on an Horiba Aqualog system following Nelson et al. 2015.

Metabolite Extraction. Exactly 800mL of the 0.22 μm filtrate was adjusted to pH 2 with trace metal grade HCl and extracted through 200 mg bed mass Bond Elut-PPL resin in a 3 mL volume cartridge (Agilent 12105005) following Petras et al.(2018). Briefly, PPL cartridges were soaked overnight with LC-MS grade methanol and pre-washed with 2x fillings of methanol, 2x LC-MS grade water and 1x methanol and dried prior to fieldwork. Immediately prior to sample extraction in the field, PPL cartridges were activated with 1x methanol and conditioned with 2x pH 2 LC-MS grade water. Samples were extracted using a peristaltic multi-channel pump with a flow rate of 8ml/min through acid-leached and sample flushed platinum-cured silicone transfer tubing and santoprene pumping tubing. Columns were desalinated with 3x cartridge fillings of pH2 LC-MS grade water and dried under N2 gas until complete color change of the resin. PPL cartridges were stored at -80 °C until elution with 2ml LC-MS grade methanol into glass vials. Extracts were dried in a Centrivap, re-dissolved in 80% MeOH:H20 with 1% formic acid and transferred into 300ul glass inserts. Extraction efficiencies were measured by evaporating solvent from a known fraction of the sample, re-dissolving in MilliQ water and analyzing for DOC on a Shimadzu TOC-V as described above, then comparing derived concentrations eluted from the PPL resin with those in the initial samples. Extraction efficiencies were then calculated by comparing the values to the DOC concentrations of the initial sample.

Liquid Chromatography Tandem Mass Spectrometry (LC-MS/MS). An aliquot (5 µL) of each sample was injected into a Vanquish ultra-high performance liquid chromatography system (UHPLC) coupled to a Q-Exactive Orbitrap Mass Spectrometer (Thermo Fisher Scientific, Bremen, Germany). Chromatographic separation was performed with a C18 core-shell column (Kinetex, 150 × 2 mm, 1.8 µm particle size, 100 Å pore size, Phenomenex, Torrance, USA) with a flowrate of 0.5 mL min^{-1} (Solvent A: H2O + 0.1% formic acid (FA), Solvent B: Acetonitrile + 0.1% FA). After injection, the samples were eluted for ten minutes, including 30 seconds of 5% Solvent B, across a linear gradient over 7.5 minutes of 5 to 50% Solvent B and 2 minutes 50 to 99% Solvent B, followed by a 2 min washout phase at 99% B and a 3 min re-equilibration phase at 5% B; ion data was collected through the washout phase. Carry over was controlled by MeOH blank injections between every 5 samples and mass and retention time accuracy and drift were monitored with a quality control mix consisting of 6 standard compounds (Sulfamethazine, Sulfamethizole, Sulfachloropyridazine, Sulfadimethoxine, Amitryptiline, Coumarin-314). Retention time and mass accuracy shifts were negligible over the course of this continuous multi-day run, manually monitored by running the quality control mix at start, midway and end and re-calibrating the mass spectrometer. Mass spectra (MS1) were acquired in positive electrospray ionization (ESI+) mode and parameters were set to 52 AU sheath gas flow, 14 AU auxiliary gas flow, 0 AU sweep gas flow and 400◦C auxiliary gas temperature. The spray voltage was set to 3.5 kV and the inlet capillary temperature was set to 320◦ C. The stacked-ring ion guide (S-Lens) was set to 50V. The maximum ion injection time was set to 100 ms with automated gain control (AGC) targets set to 1.0E6 for survey scans and 3.0E5 for MS/MS with minimum threshold of 10% C-trap AGC. Scan range for MS1 was set to 150–1,500 m/z with a m/z 200 resolution ($R_{m/z\ 200}$) of 70,000 with one micro-scan. MS/MS spectra were recorded in data dependent acquisition (DDA) mode, with precursor selection windows set to m/z 1 and up to 5 MS/MS scans of the most abundant ions per duty cycle measured with $R_{m/z\ 200}$ of 17,500 with one micro-scan. Normalized collision energy was set to a stepwise increase from 20 to 30 to 40% with $z = 1$ as default charge state. MS/MS experiments were triggered at the apex of peaks within 2–15 s from their first occurrence. Dynamic exclusion was set to 5 s. Ions with unassigned charge states were excluded from DDA as well as isotope peaks.

Calculations of Nominal Oxidation State of Carbon and Gibbs energies of oxidation half reactions. The NOSC in an organic compound is first derived from the following oxidation half reaction:

$C_aH_bN_cO_dP_eS_f^Z + (3a + 4e - d)H_2O \rightarrow a\ HCO_3^- + cNH_4^+ + eHPO_4^{2-} + fHS^- + (5a + b - 4c - 2d + 7e - f)\ H^+ + (-Z + 4a + b - 3c - 2d + 5e - 2f)\ e^-$

where Z corresponds to the net charge of the organic compound and the subscripts a, b, c, d, e and f refer to the stoichiometric numbers of the elements C, H, N, O, P and S. For this set of data a net charge of 0 was assumed for all molecular formulas derived from ZODIAC. NOSC can then be calculated from the number of electrons transferred in the half reaction (n_{e^-}) and the number of carbon atoms in the compound (a):

$$\mathrm{NOSC} = -\frac{\mathrm{ne-}}{a} + 4$$

This equation can then be expanded using the electron stoichiometry from the previous reaction

$$\mathrm{NOSC} = -\frac{-Z+4a+b-3c-2d+5e-2f}{a} + 4$$

to allow for the calculation of the NOSC in any organic compound. By then comparing the Standard Gibbs energies of the oxidation half reactions ($\Delta G^0_{\mathrm{Cox}}$) of various organic compounds to their NOSC in the compounds, LaRowe and Van Cappellen established an empirical relationship of

$\Delta G^0_{\mathrm{Cox}}$ = 60.3 – 28.5 x NOSC at 25°C and 1 bar with a with a correlation coefficient R of 0.94.

Statistical Analyses. Before subsequent statistical analyses, all exudate features were relativized by dividing peak area intensity for each exudate feature by the total exudate peak area intensity within each sample to account for minor variation in DOM loading and retention. For linear modeling statistical analyses, relative abundances were angular transformed (asin(sqrt(X)) and raw peak areas (XIC) were $\log_{10}$ transformed after adding a baseline gapfilling threshold constant 1E3. To test entire subnetworks for relative enrichment in exometabolomes we ran ANOVA (FDR $p < 0.05$) comparing the mean summed relative abundance of exudate features in each network among endpoint daytime samples, then applied a Dunnet's *post hoc* test to identify those networks significantly enriched relative to and with a mean at least twice that of the Controls. Means of various biogeochemical parameters were compared among treatments and day/night incubations using 2-way ANOVA and linear relationships between continuous

variables were done with least squares regression after transformations to approximate gaussian distributions. Where appropriate *post hoc* testing was applied as described, and False Discovery Rate (FDR) adjustments were used to control for multiple comparisons (Benjamini and Hochberg 1995). Multivariate ordinations were used for data visualization where indicated (Principal Components Analysis, Multidimensional Scaling) and unsupervised hierarchical clustering with Ward's Minimum Variance method was applied for grouping of features according to covariance patterns. Permutational ANOVA was run on bray-curtis distance matrices constructed from angular transformed feature relative abundances using the adonis function in the vegan package (Oksanen et al. 2007) of the R statistical programming environment (v3.6.3).

Data and Code Sharing. All raw and processed LC-MS/MS data as well as processed MS/MS files and feature table is available through the Mass Spectrometry Interactive Virtual Environment (MassIVE) repository (massive.ucsd.edu) with accession number MSV000082083. Software code for the ion-identity networking enabled version of MZmine2 is available at https://github.com/robinschmid/mzmine2/releases/. Molecular Networking Data and all results of the Spectra Library Comparison can be found at the Global Natural Product Social Molecular Networking (GNPS) website with the link https://gnps.ucsd.edu/ProteoSAFe/status.jsp?task=d7373e2add2a4e72a871a60c96fbbbc3. Subnetwork classification via MolNetEnhancer using Library matches and NAP results are accessioned in GNPS with the link https://gnps.ucsd.edu/ProteoSAFe/status.jsp?task=f93cb26f95d94a0c84757dd0eb0bb408. Background, transient and ambient peak identification and removal, relativization and transformation processing of mass spectral data for statistics were done using an in-house generated R script accessioned from GitHub via Zenodo (DOI: 10.5281/zenodo.3959803; Most updated dataset and data processing script can be found at: https://github.com/Zquinlan/Distinguishing-the-molecular-diversity-and-energetic-potential-of-coral-and-algal-exometabolomes)

C. E. Nelson, *et al.*, Fluorescent dissolved organic matter as a multivariate biogeochemical tracer of submarine groundwater discharge in coral reef ecosystems. *Marine Chemistry* **177**, 232–243 (2015).

K. Grashoff, M. Ehrhardt, K. Kremling, *Methods of seawater analysis, 2nd edn. Verlag Chemie* (Weinheim, 1983).

R. Kérouel, A. Aminot, Fluorometric determination of ammonia in sea and estuarine waters by direct segmented flow analysis. *Marine Chemistry* **57**, 265–275 (1997).

J. Murphy, J. P. Riley, A modified single solution method for the determination of phosphate in natural waters. *Analytica chimica acta* **27**, 31–36 (1962).

C. A. Carlson, *et al.*, Dissolved organic carbon export and subsequent remineralization in the mesopelagic and bathypelagic realms of the North Atlantic basin. *Deep Sea Research Part II: Topical Studies in Oceanography* **57**, 1433–1445 (2010).

D. Petras, *et al.*, High-resolution liquid chromatography tandem mass spectrometry enables large scale molecular characterization of dissolved organic matter. *Frontiers in Marine Science* **4**, 405 (2017).

Y. Benjamini, Y. Hochberg, Controlling the false discovery rate: A practical and powerful approach to multiple testing. J. R. Stat. Soc. Series B Stat Methodol 57, 289–300 (1995).

J. Oksanen, *et al.*, The vegan package. *Community ecology package* **10**, 719 (2007).

Sub-Network	Total Features	Exometabolite Features	Library Match Features	Porites Mean	Pocillopora Mean	CCA Mean	Dictyota Mean	Turf Mean	Control Mean	Enrichment Category	ClassyFire Class
924	11	8	0	0.0230%	0.0959%	**0.2735%**	**0.2549%**	**0.5585%**	0.0144%	All Algae	Lupin alkaloids
231	10	6	0	0.0082%	0.0026%	**0.0217%**	**0.0243%**	**0.0748%**	0.0029%	All Algae	Prenol lipids
174	7	5	1	0.0117%	0.0078%	**0.0344%**	**0.0326%**	**0.0523%**	0.0083%	All Algae	Furofurans
1409	16	5	5	0.3229%	0.2407%	**0.4331%**	**0.4743%**	**0.7290%**	0.1055%	All Algae	Prenol lipids
2876	2	2	0	0.0025%	0.0026%	**0.0223%**	**0.0103%**	**0.0201%**	0.0020%	All Algae	Amino acids and derivatives
2933	2	2	0	0.0094%	0.0053%	**0.0412%**	**0.0196%**	**0.0514%**	0.0045%	All Algae	Amino acids and derivatives
3292	3	2	0	0.0033%	0.0004%	**0.0177%**	**0.0100%**	**0.0334%**	0.0001%	All Algae	Indoles and derivatives
3327	4	2	0	0.0017%	0.0021%	**0.0087%**	**0.0098%**	**0.0062%**	0.0013%	All Algae	Organooxygen compounds
669	3	2	0	0.0022%	0.0005%	**0.0064%**	**0.0051%**	**0.0152%**	0.0000%	All Algae	Prenol lipids
1701	2	2	0	0.0128%	0.0053%	**0.0261%**	**0.0450%**	**0.0474%**	0.0078%	All Algae	Prenol lipids
3105	2	2	0	0.0052%	0.0020%	**0.0182%**	**0.0330%**	**0.0220%**	0.0035%	All Algae	Pyridines and derivatives
4468	1	1	1	0.0016%	0.0023%	**0.0074%**	**0.0090%**	**0.0148%**	0.0009%	All Algae	Steroids and steroid derivatives
1314	25	20	0	0.0010%	0.0001%	**1.3608%**	0.0072%	0.0050%	0.0016%	CCA	Macrolactams
1876	19	13	2	0.0454%	0.0184%	**0.2085%**	0.0014%	0.0214%	0.0037%	CCA	Sphingolipids
99	10	7	2	0.0150%	0.0126%	**0.0208%**	0.0038%	0.0139%	0.0020%	CCA	Glycerophospholipids
369	6	6	0	0.0005%	0.0000%	**0.0557%**	0.0022%	0.0029%	0.0006%	CCA	Steroids and steroid derivatives
930	11	4	0	0.0002%	0.0001%	**0.0285%**	0.0001%	0.0009%	0.0000%	CCA	Organooxygen compounds
1664	6	3	0	0.0141%	0.0072%	**0.0185%**	0.0083%	0.0133%	0.0138%	CCA	Fatty Acyls
1619	3	2	0	0.0108%	0.0075%	**0.0703%**	0.0059%	0.0223%	0.0038%	CCA	Amino acids and derivatives
1758	8	2	0	0.0095%	0.0091%	**0.0844%**	0.0566%	0.0668%	0.0098%	CCA	Organonitrogen compounds
5898	2	2	0	0.0001%	0.0000%	**0.0046%**	0.0002%	0.0002%	0.0001%	CCA	Piperidines
1243	2	2	0	0.0000%	0.0000%	**0.0073%**	0.0000%	0.0000%	0.0000%	CCA	Prenol lipids
3643	3	2	1	0.0001%	0.0001%	**0.0030%**	0.0001%	0.0004%	0.0002%	CCA	Unsaturated hydrocarbons
1571	2	1	0	0.0037%	0.0073%	**0.0119%**	0.0046%	0.0006%	0.0016%	CCA	Amino acids and derivatives
4334	1	1	0	0.0001%	0.0000%	**0.0013%**	0.0000%	0.0000%	0.0000%	CCA	Benzodiazepines
4050	2	1	0	0.0151%	0.0132%	**0.0295%**	0.0072%	0.0127%	0.0033%	CCA	Dihydroisoquinolines
7124	2	1	2	0.0286%	0.0329%	**0.0469%**	0.0341%	0.0172%	0.0080%	CCA	Indoles and derivatives
5708	2	1	0	0.0002%	0.0013%	**0.0072%**	0.0003%	0.0024%	0.0000%	CCA	Isoindoles and derivatives
4093	1	1	0	0.0000%	0.0002%	**0.0010%**	0.0000%	0.0000%	0.0000%	CCA	Organooxygen compounds
4629	2	1	0	0.0012%	0.0003%	**0.0116%**	0.0006%	0.0041%	0.0001%	CCA	Organooxygen compounds
457	2	1	1	0.0081%	0.0038%	**0.0060%**	0.0043%	0.0069%	0.0119%	CCA	Piperidines
1421	3	1	0	0.0009%	0.0004%	**0.0174%**	0.0004%	0.0003%	0.0009%	CCA	Prenol lipids
21	124	69	19	**1.2095%**	**2.4281%**	0.3364%	0.9579%	0.3055%	0.1496%	Corals	Fatty Acyls
80	25	10	0	**0.0603%**	**0.1613%**	0.0258%	0.0118%	0.0196%	0.0561%	Corals	Isoindoles and derivatives
375	30	8	1	**0.4024%**	**0.9654%**	0.0980%	0.1176%	0.1830%	0.0832%	Corals	Organooxygen compounds
923	13	7	5	**0.4254%**	**0.9571%**	0.2444%	0.1045%	0.2771%	0.0292%	Corals	Catechins
343	12	5	1	**0.1516%**	**0.1940%**	0.0206%	0.0090%	0.0225%	0.0137%	Corals	Macrolactams
4520	4	2	0	**0.0393%**	**0.0688%**	0.0108%	0.0042%	0.0185%	0.0042%	Corals	Coumarins and derivatives
2328	2	2	0	**0.0118%**	**0.0240%**	0.0019%	0.0042%	0.0036%	0.0005%	Corals	Diazanaphthalenes
2955	2	2	0	**0.0138%**	**0.0282%**	0.0033%	0.0021%	0.0040%	0.0008%	Corals	Macrolides and analogues
2938	2	1	0	**0.0117%**	**0.0126%**	0.0008%	0.0001%	0.0000%	0.0000%	Corals	Angucyclines
1968	2	1	0	**0.0032%**	**0.0037%**	0.0024%	0.0021%	0.0026%	0.0009%	Corals	Imidazopyrimidines
4993	2	1	0	**0.0381%**	**0.0416%**	0.0228%	0.0133%	0.0142%	0.0061%	Corals	Indoles and derivatives
148	43	10	0	4.1434%	2.3715%	3.5906%	2.1487%	2.5012%	5.8855%	Cosmopolitan	Carboxylic acids and derivatives
393	35	9	22	1.0206%	0.8999%	2.0772%	1.6094%	1.4520%	2.5557%	Cosmopolitan	Prenol lipids
1018	10	8	0	0.0047%	**0.1145%**	**0.0307%**	0.0009%	0.0067%	0.0034%	Cosmopolitan	Benzene and substituted derivatives
61	48	8	0	0.5134%	0.4112%	0.2122%	0.1202%	0.2037%	0.2957%	Cosmopolitan	Flavonoids
129	18	8	0	0.0103%	0.0017%	**0.0344%**	**0.0234%**	0.0193%	0.0027%	Cosmopolitan	Prenol lipids
601	20	5	0	0.0292%	0.0788%	**0.1611%**	0.1324%	**0.2093%**	0.0264%	Cosmopolitan	Organooxygen compounds
295	23	5	0	0.0830%	0.0686%	0.0521%	0.0280%	0.0533%	0.1469%	Cosmopolitan	Tannins
1827	9	4	4	0.0842%	0.0141%	**0.3376%**	0.0980%	**0.8309%**	0.0225%	Cosmopolitan	Heteroaromatic compounds
505	24	4	7	0.1542%	0.1487%	0.3453%	0.2579%	0.3586%	0.1129%	Cosmopolitan	Purine nucleosides
485	6	4	0	**0.0030%**	**0.0041%**	**0.0016%**	**0.0021%**	**0.0023%**	0.0000%	Cosmopolitan	Steroids and steroid derivatives
1993	3	3	0	0.0058%	0.0070%	0.0024%	0.0000%	0.0031%	0.0014%	Cosmopolitan	Benzene and substituted derivatives
1777	8	3	0	0.0008%	0.0006%	**0.0065%**	0.0014%	**0.0044%**	0.0010%	Cosmopolitan	Imidazopyrimidines
138	3	3	0	0.0010%	0.0006%	**0.0178%**	0.0016%	**0.0078%**	0.0005%	Cosmopolitan	Imidazopyrimidines
1075	16	3	0	0.0871%	0.0842%	0.0709%	0.0563%	0.0408%	0.0661%	Cosmopolitan	Isoindoles and derivatives
3585	6	3	1	0.0157%	0.0282%	**0.0926%**	0.0271%	**0.4111%**	0.0074%	Cosmopolitan	Organonitrogen compounds
81	14	3	0	0.0091%	0.6704%	0.0125%	0.0076%	0.4122%	0.0017%	Cosmopolitan	Organooxygen compounds
293	5	3	0	0.0127%	0.0206%	**0.0622%**	0.0529%	**0.1127%**	0.0120%	Cosmopolitan	Pyridines and derivatives
1766	2	2	0	0.0011%	0.0000%	**0.0090%**	0.0018%	**0.0284%**	0.0000%	Cosmopolitan	2-arylbenzofuran flavonoids
2204	2	2	0	0.0004%	0.0001%	0.0002%	0.0003%	0.0000%	0.0015%	Cosmopolitan	Amino acids and derivatives
1295	14	2	0	0.1643%	0.2422%	0.1402%	0.7848%	0.0612%	0.1274%	Cosmopolitan	Azepanes
2586	2	2	1	0.0008%	0.0016%	**0.0452%**	0.0010%	**0.0238%**	0.0000%	Cosmopolitan	Benzene and substituted derivatives
5176	3	2	0	0.0035%	0.0025%	**0.0214%**	0.0061%	**0.0268%**	0.0030%	Cosmopolitan	Benzofurans
2610	4	2	0	0.0093%	0.0028%	0.0060%	0.0078%	0.0050%	0.0259%	Cosmopolitan	Carboxylic acids and derivatives
2829	2	2	0	0.0001%	0.0000%	**0.0030%**	0.0003%	**0.0012%**	0.0001%	Cosmopolitan	Carboxylic acids and derivatives
2436	6	2	0	0.0037%	0.0112%	**0.0462%**	0.0249%	**0.0656%**	0.0038%	Cosmopolitan	Coumarins and derivatives
798	4	2	0	0.0365%	**0.0605%**	0.0418%	**0.0816%**	**0.0870%**	0.0168%	Cosmopolitan	Fatty Acyls
2054	2	2	0	0.0000%	**0.0005%**	**0.0023%**	0.0000%	0.0001%	0.0000%	Cosmopolitan	Fatty Acyls
508	2	2	1	0.0000%	0.0001%	**0.0004%**	0.0003%	**0.0016%**	0.0000%	Cosmopolitan	Fatty Acyls
2998	3	2	0	0.0025%	0.0039%	0.0009%	0.0004%	0.0008%	0.0027%	Cosmopolitan	Organooxygen compounds
2599	3	2	0	0.0091%	0.0035%	0.0044%	0.0023%	0.0022%	0.0063%	Cosmopolitan	Organooxygen compounds
1343	4	2	0	0.0215%	0.0267%	0.0108%	0.0143%	0.0319%	0.0174%	Cosmopolitan	Prenol lipids
1978	5	2	1	0.0721%	0.3518%	0.0214%	0.0353%	0.0184%	0.1632%	Cosmopolitan	Prenol lipids
1984	2	2	0	**0.0232%**	**0.0284%**	**0.0061%**	0.0021%	0.0034%	0.0001%	Cosmopolitan	Pyrans
3993	2	2	0	0.0020%	0.0020%	0.0014%	0.0026%	0.0008%	0.0032%	Cosmopolitan	Pyridines and derivatives
13	36	2	0	0.0680%	0.0630%	0.0350%	0.0615%	0.0809%	0.0817%	Cosmopolitan	Steroids and steroid derivatives

Sub-Network	Total Features	Exometabolite Features	Library Match Features	Porites Mean	Pocillopora Mean	CCA Mean	Dictyota Mean	Turf Mean	Control Mean	Enrichment Category	ClassyFire Class
302	5	2	1	0.0496%	0.0274%	0.2273%	0.0870%	0.1449%	0.0525%	Cosmopolitan	Stilbenes
813	5	2	0	0.0002%	0.0000%	0.0016%	0.0001%	0.0001%	0.0002%	Cosmopolitan	Unsaturated hydrocarbons
3044	2	1	0	0.0021%	0.0005%	0.0010%	0.0017%	0.0009%	0.0021%	Cosmopolitan	Amino acids and derivatives
3314	1	1	0	**0.0014%**	0.0008%	**0.0059%**	0.0010%	0.0010%	0.0001%	Cosmopolitan	Amino acids and derivatives
6331	2	1	0	0.0092%	0.0003%	**0.0110%**	0.0013%	**0.0228%**	0.0001%	Cosmopolitan	Azaspirodecane derivatives
685	1	1	0	0.0003%	0.0004%	0.0003%	0.0022%	0.0001%	0.0010%	Cosmopolitan	Azoles
1099	3	1	1	0.0082%	0.0048%	0.0089%	0.0057%	0.0171%	0.0123%	Cosmopolitan	Benzene and substituted derivatives
3745	2	1	1	**0.0049%**	0.0021%	**0.0070%**	**0.0076%**	**0.0318%**	0.0002%	Cosmopolitan	Benzodiazepines
88	6	1	1	0.0060%	0.0033%	0.0043%	0.0026%	0.0015%	0.0086%	Cosmopolitan	Carboxylic acids and derivatives
351	3	1	3	0.0337%	**0.0792%**	0.0469%	0.0240%	**0.0669%**	0.0149%	Cosmopolitan	Carboxylic acids and derivatives
886	2	1	0	0.0004%	0.0000%	0.0025%	0.0021%	0.0009%	0.0007%	Cosmopolitan	Diazinanes
490	17	1	0	0.0938%	0.0847%	0.0934%	0.1344%	0.1824%	0.1153%	Cosmopolitan	Fatty Acyls
1720	3	1	0	0.0337%	0.0074%	0.0171%	0.0925%	0.0115%	0.0423%	Cosmopolitan	Fatty Acyls
3808	2	1	1	0.0109%	0.0179%	0.1303%	0.0645%	0.1080%	0.0347%	Cosmopolitan	Organooxygen compounds
2441	1	1	0	0.0008%	0.0006%	0.0007%	0.0006%	0.0004%	0.0011%	Cosmopolitan	Phenol esters
3760	2	1	0	0.0002%	0.0002%	0.0001%	0.0113%	0.0081%	0.0003%	Cosmopolitan	Piperidines
116	2	1	2	**0.0002%**	0.0000%	**0.0002%**	0.0000%	**0.0002%**	0.0001%	Cosmopolitan	Piperidines
3815	1	1	0	0.0007%	0.0023%	0.0008%	0.0119%	0.0011%	0.0033%	Cosmopolitan	Prenol lipids
4839	1	1	0	0.0040%	0.0168%	0.0005%	0.0010%	0.0013%	0.0027%	Cosmopolitan	Prenol lipids
1711	1	1	0	0.0000%	0.0001%	0.0001%	0.0000%	0.0000%	0.0008%	Cosmopolitan	Prenol lipids
134	2	1	1	0.0098%	0.0077%	0.0192%	0.0303%	0.0214%	0.0117%	Cosmopolitan	Prenol lipids
2670	3	1	0	0.0131%	0.0136%	0.0922%	0.1601%	0.1982%	0.0165%	Cosmopolitan	Prenol lipids
5982	2	1	1	0.0172%	0.0133%	0.0158%	0.0199%	0.0276%	0.0140%	Cosmopolitan	Prenol lipids
3243	1	1	0	0.3100%	0.0151%	**0.4672%**	0.0766%	**0.7294%**	0.0177%	Cosmopolitan	Pyridines and derivatives
5420	2	1	0	0.0007%	0.0007%	**0.0015%**	**0.0019%**	0.0006%	0.0004%	Cosmopolitan	Pyrrolines
3620	2	1	0	0.0007%	0.0004%	0.0009%	0.0007%	0.0011%	0.0015%	Cosmopolitan	Pyrrolizidines
4796	2	1	0	0.0012%	0.0003%	0.0006%	0.0020%	0.0005%	0.0015%	Cosmopolitan	Steroids and steroid derivatives
1292	1	1	0	0.0171%	0.0060%	0.0188%	0.0036%	0.0065%	0.0239%	Cosmopolitan	Steroids and steroid derivatives
987	2	1	2	0.1307%	0.0963%	0.1017%	0.2273%	0.2169%	0.0667%	Cosmopolitan	Tetrahydrofurans
338	6	1	0	0.0211%	0.0085%	0.0226%	0.0473%	0.0387%	0.0194%	Cosmopolitan	Tetrahydroisoquinolines
125	2	1	0	0.0296%	0.0128%	0.0418%	0.0077%	0.0075%	0.0659%	Cosmopolitan	Thioethers
1248	2	1	0	0.0022%	0.0016%	0.0016%	0.0013%	0.0013%	0.0019%	Cosmopolitan	
1579	2	1	0	0.0336%	0.0158%	0.0120%	0.0048%	0.0083%	0.0584%	Cosmopolitan	
165	73	40	4	0.3475%	0.3351%	0.3858%	**4.2849%**	0.3930%	0.6745%	Dictyota	Fatty Acyls
627	15	14	8	0.1962%	0.1805%	0.2131%	**4.8883%**	0.1606%	0.3220%	Dictyota	Prenol lipids
1858	6	6	4	0.0402%	0.0828%	0.1635%	**3.7354%**	0.1127%	0.1378%	Dictyota	Steroids and steroid derivatives
474	5	4	0	0.0147%	0.0156%	0.0236%	**0.1597%**	0.0247%	0.0058%	Dictyota	Carboxylic acids and derivatives
4897	4	4	0	0.0023%	0.0099%	0.0042%	**0.2363%**	0.0013%	0.0051%	Dictyota	Coumarins and derivatives
2225	6	3	0	0.0098%	0.0095%	0.0090%	**0.1290%**	0.0493%	0.0198%	Dictyota	Amino acids and derivatives
1362	3	3	0	0.0048%	0.0062%	0.0088%	**0.0257%**	0.0047%	0.0048%	Dictyota	Lupin alkaloids
3999	3	3	3	0.0066%	0.0072%	0.0340%	**0.2765%**	0.0103%	0.0132%	Dictyota	Prenol lipids
4669	3	3	0	0.0066%	0.0030%	0.0066%	**0.1309%**	0.0073%	0.0079%	Dictyota	Steroids and steroid derivatives
396	3	2	0	0.0015%	0.0021%	0.0022%	**0.3304%**	0.0033%	0.0027%	Dictyota	Amino acids and derivatives
2407	3	2	0	0.0010%	0.0011%	0.0013%	**0.0156%**	0.0025%	0.0009%	Dictyota	Amino acids and derivatives
541	4	2	0	0.0017%	0.0016%	0.0009%	**0.0053%**	0.0016%	0.0004%	Dictyota	Benzene and substituted derivatives
4981	2	2	0	0.0005%	0.0001%	0.0003%	**0.0033%**	0.0005%	0.0002%	Dictyota	Carboxylic acids and derivatives
3900	2	2	0	0.0002%	0.0002%	0.0002%	**0.0126%**	0.0001%	0.0000%	Dictyota	Cinnamic acids and derivatives
1882	2	2	0	0.0002%	0.0003%	0.0003%	**0.0117%**	0.0002%	0.0006%	Dictyota	Diazines
5439	2	2	2	0.0071%	0.0068%	0.0055%	**0.4624%**	0.0069%	0.0039%	Dictyota	Fatty Acyls
3828	2	2	2	0.0104%	0.0027%	0.0057%	**0.0541%**	0.0063%	0.0038%	Dictyota	Fatty Acyls
1174	3	2	0	0.0008%	0.0029%	0.0021%	**0.0168%**	0.0149%	0.0007%	Dictyota	Flavonoids
1743	3	2	0	0.0000%	0.0000%	0.0002%	**0.0455%**	0.0001%	0.0000%	Dictyota	Glycerophospholipids
266	2	2	0	0.0001%	0.0000%	0.0001%	**0.0189%**	0.0001%	0.0001%	Dictyota	Isoflavonoids
801	2	2	0	0.0000%	0.0000%	0.0002%	**0.0206%**	0.0000%	0.0000%	Dictyota	Isoflavonoids
4139	4	2	1	0.0084%	0.0224%	0.0316%	**0.2595%**	0.0150%	0.0157%	Dictyota	Organooxygen compounds
1577	3	2	0	0.0006%	0.0005%	0.0007%	**0.0335%**	0.0006%	0.0009%	Dictyota	Prenol lipids
6256	2	2	0	0.0161%	0.0094%	0.0124%	**0.2567%**	0.0459%	0.0144%	Dictyota	Prenol lipids
2486	3	2	0	0.0017%	0.0010%	0.0023%	**0.0274%**	0.0041%	0.0041%	Dictyota	Pyridines and derivatives
1878	3	1	0	0.0047%	0.0032%	0.0059%	**0.0394%**	0.0144%	0.0094%	Dictyota	Amino acids and derivatives
1025	2	1	0	0.0211%	0.0137%	0.0184%	**0.0636%**	0.0335%	0.0116%	Dictyota	Benzene and substituted derivatives
2846	1	1	0	0.0001%	0.0000%	0.0000%	**0.0104%**	0.0000%	0.0000%	Dictyota	Benzopyrans
996	2	1	0	0.0001%	0.0001%	0.0003%	**0.0015%**	0.0008%	0.0001%	Dictyota	Carboxylic acids and derivatives
5642	2	1	0	0.0001%	0.0002%	0.0002%	**0.0020%**	0.0000%	0.0001%	Dictyota	Coumarins and derivatives
4172	3	1	0	0.0013%	0.0007%	0.0020%	**0.0023%**	0.0019%	0.0027%	Dictyota	Coumarins and derivatives
276	2	1	0	0.0023%	0.0015%	0.0039%	**0.0878%**	0.0016%	0.0021%	Dictyota	Coumarins and derivatives
1005	1	1	0	0.0034%	0.0028%	0.0038%	**0.0095%**	0.0070%	0.0019%	Dictyota	Fatty Acyls
2651	1	1	0	0.0001%	0.0003%	0.0002%	**0.0009%**	0.0001%	0.0000%	Dictyota	Fatty Acyls
2712	1	1	0	0.0001%	0.0000%	0.0002%	**0.0454%**	0.0000%	0.0000%	Dictyota	Glycerophospholipids
6707	2	1	0	0.0481%	0.0207%	0.0767%	**0.1047%**	0.0627%	0.0082%	Dictyota	Indoles and derivatives
2186	2	1	0	0.0004%	0.0002%	0.0002%	**0.0286%**	0.0004%	0.0001%	Dictyota	Indoles and derivatives
2992	2	1	0	0.0000%	0.0000%	0.0000%	**0.0019%**	0.0001%	0.0001%	Dictyota	Isoquinolines and derivatives
159	2	1	0	0.0012%	0.0013%	0.0019%	**0.0120%**	0.0089%	0.0015%	Dictyota	Lupin alkaloids
513	3	1	0	0.0008%	0.0013%	0.0009%	**0.0072%**	0.0006%	0.0009%	Dictyota	Macrolactams
3596	3	1	0	0.0008%	0.0007%	0.0013%	**0.0102%**	0.0022%	0.0010%	Dictyota	Organooxygen compounds
1803	2	1	0	0.0243%	0.0195%	0.0110%	**0.2129%**	0.0332%	0.0257%	Dictyota	Prenol lipids
2922	8	1	0	0.0015%	0.0013%	0.0025%	**0.0111%**	0.0052%	0.0019%	Dictyota	Prenol lipids
4717	2	1	0	0.0002%	0.0002%	0.0004%	**0.0020%**	0.0003%	0.0003%	Dictyota	Prenol lipids
1601	2	1	0	0.0137%	0.0122%	0.0144%	**0.0277%**	0.0117%	0.0142%	Dictyota	Prenol lipids

Sub-Network	Total Features	Exometabolite Features	Library Match Features	Porites Mean	Pocillopora Mean	CCA Mean	Dictyota Mean	Turf Mean	Control Mean	Enrichment Category	ClassyFire Class
3675	2	1	2	0.0065%	0.0059%	0.0093%	**0.0107%**	0.0060%	0.0066%	Dictyota	Prenol lipids
616	2	1	0	0.0018%	0.0010%	0.0010%	**0.0051%**	0.0011%	0.0016%	Dictyota	Prenol lipids
1128	1	1	1	0.0003%	0.0001%	0.0002%	**0.0070%**	0.0003%	0.0001%	Dictyota	Prenol lipids
5297	1	1	1	0.0025%	0.0002%	0.0056%	**0.4038%**	0.0007%	0.0025%	Dictyota	Prenol lipids
2152	4	1	0	0.0023%	0.0081%	0.0032%	**0.0399%**	0.0071%	0.0031%	Dictyota	Steroids and steroid derivatives
143	2	1	0	0.0077%	0.0164%	0.0131%	**0.0289%**	0.0112%	0.0046%	Dictyota	Tetrahydroisoquinolines
5446	1	1	0	0.0001%	0.0001%	0.0000%	**0.0018%**	0.0000%	0.0000%	Dictyota	Tetrahydroisoquinolines
398	22	8	6	0.1091%	0.1582%	0.1180%	**0.1713%**	**0.1778%**	0.0278%	Fleshy Algae	Quinolines and derivatives
4384	6	6	0	0.0016%	0.0019%	0.0032%	**0.1937%**	**0.1445%**	0.0008%	Fleshy Algae	Benzene and substituted derivatives
3535	7	6	0	0.0010%	0.0000%	0.0007%	**0.0109%**	**0.1142%**	0.0000%	Fleshy Algae	Prenol lipids
767	9	5	1	0.0134%	0.0042%	0.0294%	**0.1992%**	**0.8943%**	0.0122%	Fleshy Algae	Prenol lipids
34	9	3	0	0.0304%	0.0263%	0.0469%	**0.0581%**	**0.1135%**	0.0299%	Fleshy Algae	Benzene and substituted derivatives
7484	2	2	0	0.0031%	0.0018%	0.0033%	**0.0083%**	**0.0101%**	0.0004%	Fleshy Algae	Amino acids and derivatives
227	2	2	0	0.0014%	0.0013%	0.0038%	**0.0290%**	**0.0427%**	0.0024%	Fleshy Algae	Benzene and substituted derivatives
831	2	2	0	0.0000%	0.0000%	0.0002%	**0.0008%**	**0.0021%**	0.0000%	Fleshy Algae	Diarylheptanoids
1223	2	2	0	0.0002%	0.0005%	0.0005%	**0.0017%**	**0.0016%**	0.0001%	Fleshy Algae	Fatty Acyls
458	6	2	0	0.0373%	0.0484%	0.0528%	**0.0838%**	**0.1605%**	0.0153%	Fleshy Algae	Phenols
945	6	2	1	0.1444%	0.1669%	0.2335%	**0.3701%**	**0.3483%**	0.0278%	Fleshy Algae	Prenol lipids
2233	6	2	0	0.0145%	0.0132%	0.0325%	**0.0352%**	**0.0814%**	0.0092%	Fleshy Algae	Steroids and steroid derivatives
3363	2	1	0	0.0032%	0.0022%	0.0136%	**0.0259%**	**0.0484%**	0.0030%	Fleshy Algae	Amino acids and derivatives
6108	2	1	0	0.0022%	0.0022%	0.0074%	**0.0081%**	**0.0101%**	0.0020%	Fleshy Algae	Amino acids and derivatives
2548	4	1	0	0.0302%	0.0387%	0.0323%	**0.0836%**	**0.1062%**	0.0179%	Fleshy Algae	Fatty Acyls
1374	2	1	0	0.0108%	0.0137%	0.0135%	**0.0248%**	**0.0881%**	0.0106%	Fleshy Algae	Prenol lipids
3642	2	1	0	0.0134%	0.0031%	0.0088%	**0.0422%**	**0.0483%**	0.0030%	Fleshy Algae	Quinolines and derivatives
131	43	14	0	0.7237%	**1.0166%**	0.0648%	0.0198%	0.0260%	0.0047%	Pocillopora	Amino acids and derivatives
569	20	13	2	0.0673%	**0.2725%**	0.0488%	0.0481%	0.1041%	0.0141%	Pocillopora	Fatty Acyls
141	12	11	3	0.0350%	**1.2814%**	0.0074%	0.0030%	0.0204%	0.0000%	Pocillopora	Amino acids and derivatives
17	29	8	1	0.0948%	**0.4650%**	0.0421%	0.0342%	0.0416%	0.0350%	Pocillopora	Fatty Acyls
2035	10	8	0	0.2101%	**0.4815%**	0.0368%	0.0235%	0.0514%	0.0112%	Pocillopora	Fatty Acyls
167	30	8	0	0.4509%	**1.3721%**	0.2942%	0.3471%	0.3637%	0.3420%	Pocillopora	Fatty Acyls
212	10	8	0	0.0015%	**0.0309%**	0.0012%	0.0029%	0.0036%	0.0003%	Pocillopora	Prenol lipids
619	11	7	0	0.1088%	**1.4514%**	0.1148%	0.0333%	0.0954%	0.0050%	Pocillopora	Organooxygen compounds
1306	12	7	0	0.0751%	**0.3352%**	0.0211%	0.0133%	0.0398%	0.0064%	Pocillopora	Phenol ethers
637	11	7	0	0.0241%	**0.0767%**	0.0336%	0.0052%	0.0946%	0.0038%	Pocillopora	Prenol lipids
113	9	6	0	0.0127%	**0.1710%**	0.0058%	0.0034%	0.0034%	0.0078%	Pocillopora	Amino acids and derivatives
123	6	5	0	0.0030%	**0.0384%**	0.0000%	0.0005%	0.0003%	0.0000%	Pocillopora	Macrolides and analogues
90	16	4	0	0.0082%	**0.6562%**	0.0081%	0.0082%	0.4065%	0.0034%	Pocillopora	Benzene and substituted derivatives
857	4	4	0	0.0004%	**0.0474%**	0.0004%	0.0003%	0.0011%	0.0000%	Pocillopora	Fatty Acyls
1969	3	3	0	0.0014%	**0.0243%**	0.0016%	0.0010%	0.0019%	0.0003%	Pocillopora	Amino acids and derivatives
1433	6	3	0	0.0045%	**0.0777%**	0.0031%	0.0048%	0.0043%	0.0032%	Pocillopora	Benzopyrans
1408	4	3	0	0.0023%	**0.0610%**	0.0012%	0.0016%	0.0159%	0.0013%	Pocillopora	Carboxylic acids and derivatives
1609	4	3	0	0.0259%	**0.1354%**	0.0285%	0.0240%	0.0330%	0.0177%	Pocillopora	Dihydrofurans
2796	7	3	3	0.0759%	**0.1609%**	0.1245%	0.0023%	0.0248%	0.0032%	Pocillopora	Glycerophospholipids
2964	3	3	0	0.0000%	**0.0058%**	0.0000%	0.0000%	0.0000%	0.0000%	Pocillopora	Organooxygen compounds
2736	4	3	1	0.0014%	**0.0059%**	0.0004%	0.0012%	0.0005%	0.0006%	Pocillopora	Phenol ethers
6644	3	3	0	0.0059%	**0.0249%**	0.0170%	0.0079%	0.0063%	0.0059%	Pocillopora	Prenol lipids
199	38	3	0	0.0364%	**0.1326%**	0.0199%	0.0431%	0.0221%	0.0301%	Pocillopora	Prenol lipids
431	3	2	0	0.0009%	**0.0154%**	0.0001%	0.0002%	0.0007%	0.0004%	Pocillopora	Anthracyclines
4524	2	2	0	0.0006%	**0.0210%**	0.0002%	0.0002%	0.0004%	0.0002%	Pocillopora	Cinnamic acids and derivatives
4032	2	2	0	0.0021%	**0.0727%**	0.0002%	0.0002%	0.0006%	0.0000%	Pocillopora	Coumarins and derivatives
3153	5	2	0	0.0105%	**0.1090%**	0.0053%	0.0072%	0.0145%	0.0052%	Pocillopora	Diazanaphthalenes
4013	3	2	0	0.0285%	**0.0470%**	0.0136%	0.0129%	0.0076%	0.0145%	Pocillopora	Fatty Acyls
1594	2	2	0	0.0021%	**0.0230%**	0.0025%	0.0020%	0.0021%	0.0018%	Pocillopora	Fatty Acyls
2117	3	2	0	0.0023%	**0.0206%**	0.0000%	0.0000%	0.0000%	0.0000%	Pocillopora	Macrolactams
4953	2	2	2	0.0082%	**0.0514%**	0.0041%	0.0051%	0.0051%	0.0010%	Pocillopora	Organonitrogen compounds
2014	2	2	0	0.0222%	**0.0703%**	0.0023%	0.0035%	0.0036%	0.0021%	Pocillopora	Organooxygen compounds
1048	2	2	1	0.0018%	**0.0216%**	0.0021%	0.0003%	0.0012%	0.0004%	Pocillopora	Phenol esters
62	2	2	0	0.0012%	**0.0084%**	0.0017%	0.0006%	0.0017%	0.0000%	Pocillopora	Quinolines and derivatives
3824	2	2	0	0.0192%	**0.0537%**	0.0069%	0.0119%	0.0197%	0.0031%	Pocillopora	Tetrahydroisoquinolines
1704	10	1	0	0.0046%	**0.0348%**	0.0093%	0.0031%	0.0110%	0.0036%	Pocillopora	Amino acids and derivatives
2837	1	1	0	0.0000%	**0.0036%**	0.0000%	0.0000%	0.0000%	0.0000%	Pocillopora	Amino acids and derivatives
2957	1	1	0	0.0004%	**0.0054%**	0.0004%	0.0005%	0.0003%	0.0000%	Pocillopora	Amino acids and derivatives
4063	1	1	0	0.0003%	**0.0011%**	0.0001%	0.0000%	0.0001%	0.0000%	Pocillopora	Amino acids and derivatives
5082	1	1	0	0.0014%	**0.0165%**	0.0002%	0.0001%	0.0017%	0.0000%	Pocillopora	Amino acids and derivatives
1829	3	1	1	0.0087%	**0.0452%**	0.0040%	0.0053%	0.0050%	0.0112%	Pocillopora	Benzene and substituted derivatives
2931	1	1	0	0.0020%	**0.0160%**	0.0012%	0.0010%	0.0035%	0.0008%	Pocillopora	Benzene and substituted derivatives
3990	2	1	0	0.0033%	**0.0194%**	0.0010%	0.0024%	0.0021%	0.0004%	Pocillopora	Carboxylic acids and derivatives
172	24	1	0	0.0753%	**0.2173%**	0.0359%	0.0429%	0.0203%	0.0327%	Pocillopora	Coumarins and derivatives
4103	2	1	0	0.0010%	**0.0039%**	0.0015%	0.0011%	0.0020%	0.0009%	Pocillopora	Fatty Acyls
3416	1	1	0	0.0001%	**0.0014%**	0.0001%	0.0000%	0.0000%	0.0000%	Pocillopora	Flavonoids
1888	3	1	2	0.0216%	**0.1456%**	0.0127%	0.0091%	0.0085%	0.0094%	Pocillopora	Hydroxy acids and derivatives
3657	1	1	0	0.0005%	**0.0019%**	0.0001%	0.0002%	0.0001%	0.0000%	Pocillopora	Imidazopyrimidines
1012	10	1	0	0.0219%	**0.0561%**	0.0157%	0.0110%	0.0061%	0.0057%	Pocillopora	Keto acids and derivatives
2143	4	1	0	0.0052%	**0.0161%**	0.0015%	0.0018%	0.0035%	0.0009%	Pocillopora	Lupin alkaloids
1840	2	1	0	0.0042%	**0.0149%**	0.0023%	0.0025%	0.0033%	0.0027%	Pocillopora	Lupin alkaloids
1633	2	1	0	0.0001%	**0.0012%**	0.0001%	0.0001%	0.0001%	0.0002%	Pocillopora	Macrolides and analogues
4896	2	1	0	0.0011%	**0.0117%**	0.0005%	0.0003%	0.0027%	0.0005%	Pocillopora	Organonitrogen compounds
1841	5	1	2	0.0254%	**0.0681%**	0.0146%	0.0094%	0.0169%	0.0109%	Pocillopora	Organonitrogen compounds

Sub-Network	Total Features	Exometabolite Features	Library Match Features	Porites Mean	Pocillopora Mean	CCA Mean	Dictyota Mean	Turf Mean	Control Mean	Enrichment Category	ClassyFire Class
296	2	1	1	0.0026%	**0.0047%**	0.0010%	0.0013%	0.0015%	0.0007%	Pocillopora	Organooxygen compounds
1845	2	1	0	0.0003%	**0.0010%**	0.0000%	0.0003%	0.0000%	0.0000%	Pocillopora	Peptidomimetics
918	4	1	0	0.0011%	**0.0279%**	0.0013%	0.0014%	0.0012%	0.0018%	Pocillopora	Perylenequinones
5937	2	1	0	0.0010%	**0.0267%**	0.0030%	0.0057%	0.0044%	0.0016%	Pocillopora	Phenols
4212	2	1	0	0.0000%	**0.0274%**	0.0000%	0.0000%	0.0028%	0.0000%	Pocillopora	Prenol lipids
3458	2	1	0	0.0011%	**0.0049%**	0.0021%	0.0003%	0.0006%	0.0007%	Pocillopora	Prenol lipids
1569	2	1	0	0.0034%	**0.0083%**	0.0006%	0.0000%	0.0007%	0.0002%	Pocillopora	Prenol lipids
3751	2	1	1	0.0165%	**0.1397%**	0.0082%	0.0159%	0.0524%	0.0118%	Pocillopora	Prenol lipids
4296	1	1	0	0.0000%	**0.0010%**	0.0000%	0.0000%	0.0000%	0.0000%	Pocillopora	Prenol lipids
3316	1	1	0	0.0001%	**0.0008%**	0.0001%	0.0000%	0.0000%	0.0000%	Pocillopora	Triazolopyrimidines
2896	2	1	0	0.0000%	**0.0275%**	0.0000%	0.0000%	0.0001%	0.0000%	Pocillopora	
1142	7	2	0	**0.0121%**	0.0050%	0.0038%	0.0010%	0.0011%	0.0025%	Porites	Organonitrogen compounds
2334	3	1	0	**0.0762%**	0.0197%	0.0186%	0.0035%	0.0137%	0.0023%	Porites	Indoles and derivatives
980	14	1	0	**0.0792%**	0.0376%	0.0056%	0.0126%	0.0115%	0.0229%	Porites	Prenol lipids
3202	2	1	0	**0.0271%**	0.0089%	0.0031%	0.0000%	0.0087%	0.0002%	Porites	
756	40	35	0	0.3063%	0.0032%	0.0839%	0.0035%	**9.4432%**	0.0021%	Turf	Azaspirodecane derivatives
190	20	8	0	0.0681%	0.0085%	0.1210%	0.0782%	**0.1575%**	0.1609%	Turf	Fatty Acyls
87	45	6	0	0.3852%	0.4923%	0.6348%	0.7172%	**1.1351%**	0.1677%	Turf	Steroids and steroid derivatives
2983	5	4	0	0.0005%	0.0003%	0.0008%	0.0039%	**0.1694%**	0.0031%	Turf	Benzene and substituted derivatives
1198	7	4	0	0.0109%	0.0089%	0.0222%	0.0182%	**0.0675%**	0.0033%	Turf	Coumarins and derivatives
3025	7	4	0	0.0090%	0.0092%	0.0131%	0.0124%	**0.0373%**	0.0091%	Turf	Quinolines and derivatives
1414	4	3	0	0.0012%	0.0004%	0.0003%	0.0002%	**0.0335%**	0.0003%	Turf	Azaspirodecane derivatives
1907	4	3	0	0.0000%	0.0000%	0.0001%	0.0001%	**0.0111%**	0.0000%	Turf	Benzene and substituted derivatives
1443	5	3	0	0.0080%	0.0054%	0.0222%	0.0261%	**0.0532%**	0.0016%	Turf	Fatty Acyls
1980	4	3	0	0.0002%	0.0001%	0.0002%	0.0037%	**0.0866%**	0.0002%	Turf	Piperidines
403	3	3	0	0.0009%	0.0003%	0.0070%	0.0015%	**0.0257%**	0.0013%	Turf	Prenol lipids
5626	2	2	0	0.0002%	0.0002%	0.0020%	0.0037%	**0.0213%**	0.0000%	Turf	Amino acids and derivatives
3143	4	2	0	0.0001%	0.0001%	0.0092%	0.0002%	**0.1205%**	0.0000%	Turf	Azepines
702	2	2	1	0.0015%	0.0078%	0.0006%	0.0064%	**0.0756%**	0.0016%	Turf	Azoles
1173	2	2	0	0.0000%	0.0000%	0.0017%	0.0012%	**0.0175%**	0.0000%	Turf	Cinnamic acids and derivatives
2157	8	2	0	0.0001%	0.0002%	0.0006%	0.0002%	**0.5450%**	0.0003%	Turf	Fatty Acyls
1202	2	2	0	0.0030%	0.0011%	0.0060%	0.0043%	**0.0138%**	0.0002%	Turf	Indoles and derivatives
2107	2	2	0	0.0000%	0.0000%	0.0003%	0.0008%	**0.0063%**	0.0003%	Turf	Isoindoles and derivatives
362	6	2	0	0.0149%	0.0056%	0.0163%	0.0224%	**0.0662%**	0.0085%	Turf	Lupin alkaloids
1786	4	2	1	0.0004%	0.0010%	0.0005%	0.0082%	**0.0820%**	0.0004%	Turf	Organonitrogen compounds
1150	2	2	0	0.0000%	0.0000%	0.0002%	0.0000%	**0.0130%**	0.0000%	Turf	Organooxygen compounds
2734	2	2	0	0.0001%	0.0000%	0.0000%	0.0011%	**0.0262%**	0.0000%	Turf	Organooxygen compounds
3846	3	2	0	0.0017%	0.0029%	0.0039%	0.0096%	**0.0574%**	0.0035%	Turf	Prenol lipids
1839	3	2	0	0.0006%	0.0002%	0.0016%	0.0031%	**0.2494%**	0.0014%	Turf	Prenol lipids
187	2	2	0	0.0000%	0.0002%	0.0001%	0.0007%	**0.0023%**	0.0000%	Turf	Prenol lipids
5298	5	2	0	0.0024%	0.0016%	0.0096%	0.0207%	**0.2920%**	0.0026%	Turf	Steroids and steroid derivatives
1332	4	2	0	0.0025%	0.0024%	0.0087%	0.0117%	**0.0197%**	0.0027%	Turf	Tetrapyrroles and derivatives
5843	3	1	0	0.0029%	0.0096%	0.0045%	0.0040%	**0.0480%**	0.0063%	Turf	Amino acids and derivatives
67	6	1	0	0.0005%	0.0006%	0.0007%	0.0054%	**0.0419%**	0.0002%	Turf	Benzene and substituted derivatives
5413	2	1	0	0.0000%	0.0000%	0.0004%	0.0001%	**0.0117%**	0.0003%	Turf	Benzene and substituted derivatives
4148	2	1	0	0.0001%	0.0001%	0.0004%	0.0000%	**0.0114%**	0.0000%	Turf	Benzopyrans
2331	1	1	0	0.0024%	0.0021%	0.0053%	0.0048%	**0.0145%**	0.0019%	Turf	Carboxylic acids and derivatives
1060	3	1	0	0.0030%	0.0028%	0.0024%	0.0016%	**0.0059%**	0.0008%	Turf	Coumarins and derivatives
3240	2	1	0	0.0049%	0.0041%	0.0118%	0.0087%	**0.0323%**	0.0040%	Turf	Diazines
440	1	1	0	0.0001%	0.0000%	0.0000%	0.0000%	**0.0043%**	0.0002%	Turf	Fatty Acyls
3706	2	1	0	0.0002%	0.0002%	0.0010%	0.0009%	**0.0115%**	0.0006%	Turf	Harmala alkaloids
4342	2	1	0	0.0002%	0.0001%	0.0022%	0.0015%	**0.0050%**	0.0004%	Turf	Macrolactams
1101	2	1	0	0.0088%	0.0170%	0.0435%	0.0375%	**0.0927%**	0.0130%	Turf	Organonitrogen compounds
5422	1	1	1	0.0000%	0.0000%	0.0000%	0.0000%	**0.0008%**	0.0000%	Turf	Phenols
5801	2	1	0	0.0037%	0.0036%	0.0037%	0.0134%	**0.0480%**	0.0002%	Turf	Prenol lipids
451	2	1	0	0.0021%	0.0036%	0.0178%	0.0151%	**0.0354%**	0.0007%	Turf	Tetrahydroisoquinolines
1372	2	1	0	0.0043%	0.0006%	0.0127%	0.0141%	**0.0567%**	0.0013%	Turf	Thiophenes

Supplementary Table S1. List of Subnetworks enriched in exometabolomes. The relative abundance of all Exudate nodes in each subnetwork containing exometabolite features were summed and statistically compared among the six endpoint treatments followed by a post hoc Dunnet's test for 2X enrichment over the control. Network mean relative abundances listed in Bold Underline are significantly enriched. Enrichment Category denotes which treatments the network was enriched in.The ClassyFire Class derived from MolNetEnhancer is listed for each network.
